# Natural Chain-Breaking Antioxidants and Their Synthetic Analogs as Modulators of Oxidative Stress

**DOI:** 10.3390/antiox10040624

**Published:** 2021-04-19

**Authors:** Vessela D. Kancheva, Maria Antonietta Dettori, Davide Fabbri, Petko Alov, Silvia E. Angelova, Adriana K. Slavova-Kazakova, Paola Carta, Valerii A. Menshov, Olga I. Yablonskaya, Aleksei V. Trofimov, Ivanka Tsakovska, Luciano Saso

**Affiliations:** 1 Institute of Organic Chemistry with Centre of Phytochemistry, Bulgarian Academy of Sciences, “Acad. G. Bonchev” Str., bl.9, 1113 Sofia, Bulgaria; adriana.kazakova@orgchm.bas.bg; 2 CNR, Istituto di Chimica Biomolecolare, Traversa La Crucca 3, 07100 Sassari, Italy; mariaantonietta.dettori@cnr.it (M.A.D.); davidegaetano.fabbri@cnr.it (D.F.); paola.carta@cnr.it (P.C.); 3 Institute of Biophysics and Biomedical Engineering, Bulgarian Academy of Sciences, “Acad. G. Bonchev” Str., bl.21, 1113 Sofia, Bulgaria; petko@biophys.bas.bg (P.A.); itsakovska@biomed.bas.bg (I.T.); 4 Institute of Optical Materials and Technologies “Acad. J. Malinowski”, Bulgarian Academy of Sciences, “Acad. G. Bonchev” Str., bl.109, 1113 Sofia, Bulgaria; 5 Emanuel Institute of Biochemical Physics, Russian Academy of Sciences, “Kosigina”4 Str., 119334 Moscow, Russia; vinoman66@mail.ru (V.A.M.); olga.yablonsky@gmail.com (O.I.Y.); avt_2003@mail.ru (A.V.T.); 6 Moscow Institute of Physics and Technology (National Research University), Institutskiy per. 9, Dolgoprudny, 141701 Moscow, Russia; 7 Department of Physiology and Pharmacology “Vittorio Erspamer”, Sapienza University, P.le Aldo Moro 5, 00185 Rome, Italy; luciano.saso@uniroma1.it

**Keywords:** oxidative stress, bio-antioxidant, chain breaking antioxidant activity, structure–activity relationship, synthesis

## Abstract

Oxidative stress is associated with the increased production of reactive oxygen species or with a significant decrease in the effectiveness of antioxidant enzymes and nonenzymatic defense. The penetration of oxygen and free radicals in the hydrophobic interior of biological membranes initiates radical disintegration of the hydrocarbon “tails” of the lipids. This process is known as “lipid peroxidation”, and the accumulation of the oxidation products as peroxides and the aldehydes and acids derived from them are often used as a measure of oxidative stress levels. In total, 40 phenolic antioxidants were selected for a comparative study and analysis of their chain-breaking antioxidant activity, and thus as modulators of oxidative stress. This included natural and natural-like *ortho*-methoxy and *ortho*-hydroxy phenols, nine of them newly synthesized. Applied experimental and theoretical methods (bulk lipid autoxidation, chemiluminescence, in silico methods such as density functional theory (DFT) and quantitative structure–activity relationship ((Q)SAR) modeling) were used to clarify their structure–activity relationship. Kinetics of non-inhibited and inhibited lipid oxidation in close connection with inhibitor transformation under oxidative stress is considered. Special attention has been paid to chemical reactions resulting in the initiation of free radicals, a key stage of oxidative stress. Effects of substituents in the side chains and in the phenolic ring of hydroxylated phenols and biphenols, and the concentration were discussed.

## 1. Introduction

Oxidative stress occurs as a result of an imbalance between the increased productions of oxidants, most prominently of a free radical nature on the one hand, and/or as a result of compromising the antioxidant protection systems in the cells of the living organism, on the other. Free radicals (FRs) play an important role in the normal course of physiological processes. Many of the chemical reactions in vivo occur with the formation of FRs. The genesis of several chronic degenerative diseases, including malignant tumors, a significant proportion of cardiovascular and neurological diseases, as well as age-related changes in the human body, are associated with oxidative stress in the cells. On the other hand, disturbances in the dynamics and site of generation of free radical products, often under the influence of extrinsic factors causing the formation of FRs, can cause various damage in cells and a wide range of pathological conditions. FRs can damage lipids in cell and intracellular membranes, stimulating lipid peroxidation processes and thus disrupt cell organelle functions. It is important to know that as a result of lipid peroxidation, secondarily-generated free radical products are produced, some of them long-living, which thereby can migrate both inside cells and circulate through the bloodstream and enter other organs, resulting in DNA damage and causing disorders in the functioning of cellular genetic apparatuses [1,2,3].

Bio-antioxidants have been used in the last years in the prevention of human diseases as components of nutritional supplements, and for the treatment of various diseases as pertinent agents in monotherapy or complex drug therapy. Antioxidant therapy is a modern method aimed at preventing or limiting the abnormal production of FRs in cells, including aggressive oxygen-containing radicals, and limiting their harmful effects on the body [4,5]. Burlakova [6] has demonstrated that the pathogenesis of most diseases is characterized by increased levels of lipid peroxidation, processes that can be controlled by antioxidants. In some diseases, especially at an early stage, the use of so-called monotherapy with high concentration antioxidants alone is effective enough. In other diseases, the use of antioxidants complements and/or enhances the therapeutic effect of essential medicines [7].

The activity of antioxidants depends on complex factors, including the nature of the antioxidants, the properties of the substrate to be oxidized, as well as the stage and type of oxidation. In this context, the following two aspects should be mentioned. The first one pertains to the antioxidant potential, determined by the antioxidant’s composition and the properties of its ingredients that are the subject of natural product chemistry and food chemistry, while the second aspect refers to the biological effects that, among other things, are governed by the bioavailability of the antioxidants, which is a medical-biological problem. Thus, antioxidants are considered essential for the body’s defense system against oxidative stress. Following this trend, during the last decade, the antioxidant properties of biologically active compounds have been the subject of growing interest [8,9,10,11].

The 40 phenolic antioxidants (nine of them newly synthesized) selected for this comparative study and analysis of their chain-breaking antioxidant activity, are natural and synthetic natural-like *ortho*-methoxy phenols, *ortho*-hydroxy phenols and their corresponding biphenyl dimers (see Table 1).

2-Methoxyphenols constitute one of the common classes of secondary metabolites in medicinal plants. When these phenols are substituted at position 4 with an electron donating group (EDG), these compounds become potential antioxidants [12]. This is due to the favored stabilization of the generated phenoxyl radical by forming a five-membered ring with the methoxy group and to the presence of the EDG group in *para* position to the phenol OH group. Hydroxylated biphenyls are widely present in nature, and unlike the corresponding natural monomeric phenols, they represent an important source of bioactive compounds that have not been well studied. Structurally, they are C2-symmetrical dimers of phenols where two aromatic rings are bridged by a single C–C bond [13].

Curcumin, a bright yellow spice derived from the rhizome of *Curcuma longa* of the ginger family (*Zingiberaceae*), displays a wide range of pharmacological activities [14] such anti-microbial, anti-malarial, anti-inflammatory, anti-proliferative, anti-angiogenic, anti-tumor and anti-aging properties. This spice has been a part of the human diet in several Asiatic countries, mainly India and China, for hundreds of years and has been used in their folk medicine for the treatment of many diseases such as diabetes, Alzheimer, cancer and rheumatoid arthritis. Curcumin is not stable under physiological conditions and its degradation products are mainly vanillin, ferulic acid and dehydrozingerone [15]. Curcumin is also a well-known natural antioxidant that can protect biomembranes against peroxidative damage, mainly as a scavenger of FRs [16]. The clinical use of curcumin is limited because of its low bioavailability, hydrophobic nature of the molecule, poor water solubility, low oral absorbability, and rapid metabolic rate. Concerning the persistence of curcumin towards oxidation, a recent study is noteworthy [17]. It has been found that curcumin is essentially inert towards autooxidation in aerated alkaline DMSO at room temperature. This is highly surprising, since curcumin is prone to rapid autooxidation in basic aqueous solutions and, furthermore, this is astonishing because in alkaline DMSO the autooxidation of an appreciable number of substances proceeds particularly fast [17]. Besides this unprecedented behavior, in such a medium curcumin exhibits also rather unusual spectral and luminescent properties [17].

The poor bioavailability and rapid metabolism of curcumin have prompted researchers to look for novel drug delivery systems (liposomes, system of nanoparticles, phospholipid complexes) and new synthetic curcumin analogs to overcome these drawbacks and gain in efficacy with reduced toxicity. In the last 10 years, many curcumin analogs have been synthesized by independent research groups. In the structure of curcumin, two guaiacyl units are linked together through a heptadiene-3,5-diketone or a heptadiene-3,5-hydroxyketone in the keto and enol form, respectively. It is commonly assumed that the two phenolic moieties are responsible for the radical scavenging properties of such molecules [13]. Structural modifications have revolved mainly around two parts of the curcumin structure: the phenyl moiety holding the hydroxylated substituents and the unsaturated carbonyl chain. These structural modifications lead to new compounds with improved stability, water solubility and bioavailability and effective antioxidant [18], antitumor [19,20], anti-inflammatory [21] and neuroprotective activities [22].

Chain-breaking antioxidant activity is the ability of compounds to shorten the oxidation chain length as a result of its reaction with peroxyl radicals. It is in contrast to radical-scavenging activity towards different free radicals, which gives information only about the H-donating propensity of the studied compounds and some preliminary information on their possibility to be used as antioxidants, and thus differs significantly from chain-breaking antioxidant activity. In this context, by antioxidant activity we mean the chain-breaking activity of the compounds [23,24,25,26].

Together with their antioxidant activity, an important aspect of the compounds intended to be used as drugs or food additives are their ADME/Tox (absorption, distribution, metabolism, excretion and toxicity) properties. Application of in silico (Q)SAR (quantitative structure–activity relationship) approaches to predict ADME/Tox is pivotal to optimize the compounds’ structure in order to achieve the desired properties [27,28].

Over the past decades, density functional theory (DFT) calculations have been greatly developed and have become an important tool in studying the properties of a wide variety of compounds and materials [29]. Nowadays, DFT calculations are routinely used for unraveling the structure–activity relationship of bioactive compounds and antioxidants [30].

The applied experimental and theoretical methods in the study (bulk lipid autoxidation, measuring chemiluminescence in model system, and DFT calculations) clarify structure–activity relationship for the selected compounds. Effects of concentration and side chain/phenolic ring substitution are discussed. Experimentally assessed chain-breaking antioxidant activities determined by bulk lipid autoxidation and chemiluminescence (CL) are compared with the activity to scavenge aggressive peroxide radicals by the HAT (hydrogen atom transfer) mechanism theoretically predicted by DFT calculations. Important ADME/Tox properties are explored using in silico methods.

The significance of this study is closely related to the improvement of the quality and increase in the oxidative stability of lipids and lipid products as well as to the social impact associated with the improved nutrition and human health.

For the purpose of this comparative study, we considered not only newly synthesized antioxidants, but also published results on similar phenolic antioxidants structurally related to them (Table 1).

A set of curcumin analogs (natural and natural-like phenolic and biphenolic compounds) has been screened for their chain-breaking antioxidant activity. The large number of compounds tested (40, 9 of them newly synthesized) in vitro and in silico yields multiple structure–activity relationships that provide useful information and ideas for further modifications of the main curcumin scaffold.

## 2. Materials and Methods

### 2.1. Instruments and Reagents

All ^1^H NMR and ^13^C NMR spectra were recorded on Varian Mercury Plus spectrometer operating at 399.93 MHz and 100.57 MHz for the ^1^H and ^13^C nucleus, respectively (Varian, Palo Alto, CA, USA). Chemical shifts δ are given in ppm and coupling constants in Hertz (Hz); multiplicities are indicated by s (singlet), d (doublet), dd (doublet of doublets), t (triplet). Shifts are given in ppm relative to the remaining signals of the deuterated solvent used. Elemental analyses were performed using an elemental analyzer model 240 C (Perkin-Elmer, Waltham, MA, USA). Flash chromatography was carried out with silica gel 60 (230–400 mesh, VWR, Milan, Italy) eluting with appropriate solution in the stated v:v proportions. Analytical thin-layer chromatography (TLC) was performed with 0.25 mm thick silica gel plates (Sigma-Aldrich, Munich, Germany). The purity of all new compounds was judged to be >98% by ^1^H-NMR spectral determination. All reagents and solvents are of commercial-grade quality and used as purchased from various producers (Merck, Milan, Italy; VWR, Milan, Italy). All solvents were of HPLC grade and were used without additional purification or drying, unless otherwise noted. Standard antioxidants Creosol (**Cr**), ferulic acid (**FA**), caffeic acid (**CA**), eugenol (**Eu**), *iso*-eugenol (**isoEu**), Vanillin (**Va**) curcumin (**Curc**) and Apocynin (**Apo**) were purchased from E. Merck (Darmstadt, Germany) and were used without further purification. Melting points were determined on a 530 apparatus (Büchi, Flawil, Switzerland) and are uncorrected.

Compounds **M5**, **D5**, **M8**, **D8**, **M9** and **D9** were prepared according to Slavova-Kazakova et al. [25]. Compounds **DApo**, **DVa**, **DCr**, **DEu** were prepared according to Koleva et al. [31]. Compounds **M3** and **D3** were prepared according to Slavova-Kazakova et al. [13]. Compounds **M1**, **D1**, **M2** and **D2** were prepared according to Marchiani et al. [32]. Compounds **DFA**, **HFA** and **DHFA** were prepared according to Russel et al. [33]. Compound **HPh** was prepared according to Wenskowsky et al. [34]. Compound **DHPh** was prepared according to Dettori et al. [35]. Compound **HCh** was prepared according to Nishiwaki et al. [36]. Compound **HCA** was prepared according to Aung et al. [37].

### 2.2. Chemical Synthesis

#### 2.2.1. General Procedure for the Synthesis of Compounds **M4**, **M6** and **M7**

The appropriate ester (ethyl acetate for **M6** and **M4;** ethyl propionate for **M7**) (6 eq) was added to a solution of the reagent (**M2** for **M4**; **M1** for **M6** and **M7**) (1 eq) and sodium ethoxide (4 eq) in tetrahydrofuran (40 mL). The solution was stirred for 12 h at room temperature (rt), acidified with hydrochloridric acid (10% solution) and extracted with ethyl acetate (3 × 50 mL). The organic phases were combined and dried over anhydrous sodium sulphate. The crude product was concentrated under reduced pressure and purified by flash chromatography using petroleum ether:ethyl acetate 1:1 as eluents to give pure **M4** or **M6** or **M7** as a 9:1 mixture of β-ketoenol and diketo tautomers.

#### 2.2.2. General Procedure for the Synthesis of Compounds **D4** and **D6**

Ethyl acetate (12 eq) was added to a solution of the reagent **(D1** for **D6** or **D2** for **D4** [32] (1 eq) and sodium ethoxide (8 eq) in tetrahydrofurane (40 mL). The solution was stirred at 70 °C for 18 h, acidified with hydrochloridric acid (10% solution) and extracted with ethyl acetate (3 × 50 mL). The organic phases were combined and dried over anhydrous sodium sulphate. The crude product was concentrated under reduced pressure and purified by flash chromatography using petroleum ether: ethyl acetate 1:1 as eluents to give pure **D4** or **D6** as a 9:1 mixture of β-ketoenol and diketo tautomers.

#### 2.2.3. 3,3′-Dimethoxy-5,5′-di((E)-prop-1-en-1-yl)-[1,1′-biphenyl]-2,2′-diol **DisoEu**

To solution of eugenol dimer **DEu** (0.22 g, 0.61 mmol) in diethylene glycol dimethyl ether (5 mL), potassium *tert*-butoxide (0.41 g, 3.66 mmol) was added. The reaction mixture was stirred under MW irradiation for 30 min at 100 °C. The crude product was diluted with dichloromethane, acidified with hydrochloridric acid (10% solution) and extracted with dichloromethane (3 × 50 mL). The organic phases were combined and dried over anhydrous sodium sulphate. The crude product was washed with a mixture of dichloromethane and petroleum ether 1.1 to give 0.10 g of **DisoEu** as a beige solid.

#### 2.2.4. 5,5′-Diallyl-[1,1′-biphenyl]-2,2′,3,3′-tetraol **DHCh**

To a solution of a **DEu** [31] (1 g, 3 mmol) in dichloromethane (30 mL) at −60 °C under nitrogen boron tribromide (1.5 g, 6 mmol) was added dropwise. The reaction mixture was stirred at −60 °C for 1 h then at rt for 12 h. The mixture was washed with water (100 mL) and extracted with ethyl acetate (2 × 20 mL). The organic solution was dried over sodium sulphate, and rotoevaporated, the crude product was washed with dichloromethane (2 × 10 mL) to give **DHCh** as a white solid.

#### 2.2.5. (2E,2′E)-3,3′-(5,5′,6,6′-Tetrahydroxy-[1,1′-biphenyl]-3,3′-diyl) diacrylic acid **DCA**

To a solution of a **DFA** [33] (0.4 g, 1 mmol) in dichloromethane (30 mL), boron tribromide (0.5 g, 2 mmol) was added dropwise under nitrogen. The reaction mixture was stirred at −60 °C for 1 h, and at rt for 12 h. The mixture was then washed with water (100 mL) and filtered to give **DCA** as a dark solid.

#### 2.2.6. 3,3′-(5,5′,6,6′-Tetrahydroxy-[1,1′-biphenyl]-3,3′-diyl) dipropanoic acid **DHCA**

Ten percentage palladium on activated carbon (0.02 g) and sodium acetate (0.19 g, 2.3 mmol), were added to **DCA** (0.2 g, 0.56 mmol) in methanol (10 mL). The heterogeneous mixture was stirred under a hydrogen atmosphere at room temperature for 3 h. The mixture was filtered through celite, which afforded **DHCA** as a brown solid.

All spectral characteristics are presented in the Supporting Information (Figures S1–S3).

### 2.3. Lipid Autoxidation

Kinetically pure (cleaned from pro- and antioxidants) triacylglycerols of sunflower oil (**TGSO**) were used as a model lipid substrate and the kinetics of lipid autoxidation was monitored by iodometric determination of peroxide value (PV). Details on the model employed to assess the antioxidant activity of the compounds under study can be found as Supporting information (SI). This model provides information about the potential of studied phenolic compounds to inhibit the lipid autoxidation process, (i.e., to react as chain-breaking antioxidants) and allows the determination of the main kinetic parameters (i.e., protection factor PF, induction period IP_A_, inhibition degree ID, initial rate of inhibited oxidation R_A_, respectively).

Basic kinetic scheme of lipid autoxidation: non-inhibited in absence of an antioxidant and inhibited in the presence of monophenolic and biphenolic antioxidants is presented in Supporting Information (Schemes S1 and S2, respectively).

#### 2.3.1. Determination of the Main Kinetic Parameters of the Studied Compounds (Details Are in Supporting Information)

Antioxidant efficiency in terms of protection factor (PF) refers to how many times the antioxidant increases the oxidation stability of the lipid sample, and can be determined as a ratio between the induction periods in the presence (IP_A_) and in the absence (IP_C_) of the antioxidant, i.e., PF = IP_A_/IP_C_.

Antioxidant reactivity in terms of inhibition degree (ID) is a measure of the antioxidant reactivity, i.e., how many times the antioxidant shortens the oxidation chain length, i.e., ID = R_C_/R_A_ (initial oxidation rate for control sample in absence of antioxidant, R_C_, and in presence of an antioxidant, R_A_).

It has been proven [9,11,23] that the efficiency of an antioxidant is generally based on the balance between the rate of inhibition reaction (k_A_) and the transfer reactions (k_-A_) and (k_p_’).

#### 2.3.2. Statistical Analysis

The measurements of three parallel lipid samples with and without studied antioxidants were done. The results have been presented as mean value ± standard deviation and compared by Student’s *t*-test (Microsoft Excel software).

### 2.4. Estimation of Rate Constant of Antioxidant Reaction with Peroxyl Radicals (k_A_) Using Kinetic Chemiluminescence (CL) Method (Details Are Provided in Supporting Information)

The chemiluminescence (CL) measurements were performed with the Hamamatsu photosensor module H7467 equipped with the RS-232C interface. The probe chemiluminescent hydrocarbon ‘cocktails’ for the assessment of the scavenging activity of an antioxidant towards peroxy radicals consisted of the chlorobenzene solutions of ethylbenzene (RH) being oxidized by molecular oxygen in the presence of 2,2′-azobisisobutyronitrile (AIBN) as free radical source applied to initiate the oxidation process. All the chemicals used in this study were obtained from commercial suppliers and purified by published procedures.

The strategy behind the chemiluminescence approach used herein is based on the peroxyl-radical-mediated excited-state generation in oxidation of hydrocarbon (RH, in the present case, ethylbenzene) [38]. The advantage of this hydrocarbon is the chemiluminescent substrate resides in a well-known mechanism of its oxidation at moderate temperatures (20–80 °C). Under these conditions, the oxidation rate is easy to control by choosing an appropriate concentration of the free radical initiator, Y in Scheme S3, presented in the Supporting Information (in this case, AIBN) [38].

### 2.5. Computational Studies

#### 2.5.1. DFT Computational Details

DFT calculations were performed using Gaussian 09 computational chemistry software package [39]. Unrestricted open-shell approach without symmetry constraints and with default convergence criteria was used in the geometry optimization of the monomers/dimers and phenoxyl radicals studied. The homolytic O−H bond dissociation enthalpies (BDEs) were computed from the results of calculations at B3LYP/6-31+G(d,p) level [40,41,42]. For B3LYP mean unsigned errors (mean absolute deviations from the reference data) reported for the BDEs are below 0.5 kcal mol^−1^. The used hybrid functional/basis set combination has proven reliable in the description of the phenoxyl radicals [43]. A check for the presence of spin contamination was performed but no spin contamination found for the systems with a multiplicity other than one (radicals). Frequency calculations for each optimized structure (monomers/dimers and respective radicals) were run at the same level of theory. No imaginary frequency was found for the lowest energy configuration of any of the optimized species. Unscaled thermal corrections to enthalpy from the vibrational frequency calculations were added to the total electronic energies. The equation BDE = H_298_(AO^•^) + E_T_(H^•^) − H_298_(AOH) was used for the calculation of the BDEs. The enthalpies H_298_(AO^•^) and H_298_(AOH) of the radical species and parent compounds, respectively, were calculated at 298 K. E_T_(H^•^), calculated total energy of H^•^, is −313.93 kcal mol^−1^. PyMOL molecular visualization system developed by W. DeLano was used for generation of the molecular graphics images [44].

#### 2.5.2. In Silico Prediction of ADME/Tox Properties

A number of ADME properties were predicted as follows:

- HIA (human intestinal absorption, 100 mg dose) prediction was based on MAD (maximum absorbable dose) as calculated by Avdeef, 2012 [45]. To calculate MAD, an in-house predictive QSAR model of PAMPA (parallel artificial membrane permeability assay) was employed [46];
- Prediction of various ADME/Tox properties using models implemented in ACD/Percepta software [47];
  - Prediction of CNS (central nervous system) access based on estimated brain/plasma equilibration rate, and steady-state brain/plasma distribution ratio.
  - Prediction of interactions with P-glycoprotein and Cytochrome P450 based on the respective models in the software platform.
- Toxicity predictions;
  - Toxicity prediction for multiple endpoints in a number of mammalian species was carried out using Derek Nexus knowledge-based expert system v.6.1.0 [48]. The system identifies alerts with a particular level of likelihood to exert a given toxic effect. The predictions in Derek Nexus are provided with the following levels of likelihood from highest to lowest order: certain, probable, plausible, equivocal, doubted, improbable, and impossible. In this study, a threshold for the level of likelihood “plausible” was applied meaning “the weight of evidence supports the proposition” [49].
  - Toxicity prediction using ACD/Percepta models with probabilistic categorization that is based on calculated of the effect occurrence and the reliability index of the prediction” [47]. The following toxicity endpoints were estimated: mutagenicity based on the AMES test model, acute toxicity, endocrine system disruption, based on estimated compound’s binding affinity to ERα (estrogen receptor alpha).

## 3. Results and Discussion

### 3.1. Synthesis of Compounds ***M4***, ***D4***, ***M6***, ***D6***, ***M7***, ***DisoEu***, ***DHCh***, ***DCA***, ***DHCA***

**M4**, **D4**, **M6**, **D6**, **M7** were prepared by acylation of the appropriate precursor (**M2**, **D2**, **M1**, **D1**) with ethylacetate or ethyl propionate and sodium ethoxide in tetrahydrofurane for 12 h at RT. The procedure involves a nucleophilic attack of the deprotonated base of α-β ketone generated in the starting methyl ketone by sodium ethoxide to the ester carboxylic carbon, loss of the ethoxy group and finally water to obtain the β-keto enol group. This procedure appears an alternative to classic preparations reported in the literature for the synthesis this class of products through the boron oxide based methodology [50]. All compounds were obtained as 9/1 mixture of β-ketoenol/diketo tautomers, showing chemical yields ranging from 58 to 85%. **DisoEu** was prepared by microwave induced double bond isomerization of **DEu** under basic conditions. MW irradiation for 30 min at 100 °C in diethylene glycol dimethyl ether and potassium *tert*-butoxide gave **DisoEu** in 50% yield. Boron tribromide in dichloromethane was used for effecting complete demethylation of aryl methyl ethers **DEu** and **DFA** at −60 °C to obtain **DHCh** and **DCA** respectively in yields ranging from 51 to 89%. Chemoselective hydrogenation of **DCA** by the use of palladium on activated carbon and sodium acetate in methanol under a hydrogen atmosphere at room temperature for 3 h gave **DHCA** in 95% yields. The saturated acid **DHCA** maintained the acid moiety of the unaltered precursor.

### 3.2. Experimental Studies of the Antioxidant Activity

#### 3.2.1. Chain-Breaking Antioxidant Activity during Bulk Lipid Autoxidation

The main kinetic parameters of *ortho*-methoxyphenols under study are summarized in Table 2.

This comparative study is based on the effects imposed by the following measures:

- side chain substitution;
- concentrations used (0.1 mm and 1.0 mM);
- presence of biphenyl structure (dimers vs. monomers);
- phenol ring substitution (catechol/guaiacol structure).

*Ortho*-methoxyphenols

⮚ *Effect of the substituents in the side chain*

**Figure 1:**
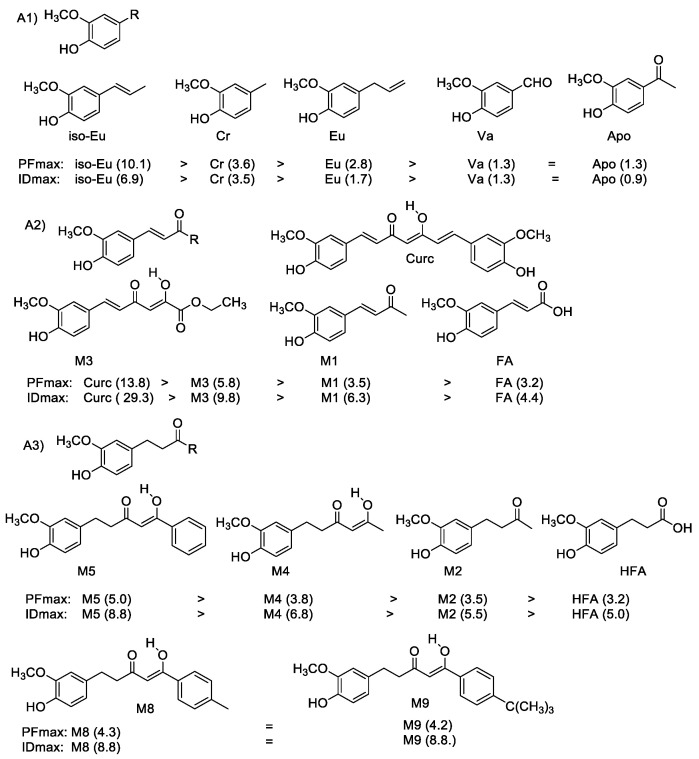
Effect of the substituents in the side chain of selected A1–A3 *ortho*-methoxyphenols expressed in terms of PF and ID values.

Comparative kinetic analysis demonstrates new orders of antioxidant activities manifested by the main kinetic parameters, namely, protection factor (PF) and inhibition degree (ID) (Figure 1).

**Monomers:**

Comparison of A1 compounds

Antioxidant efficiency and inhibition degree:

**isoEu** >> **Cr** ≥ **Eu** > **Va** = **Apo** (**isoEu** is the strongest antioxidant)

The A1 group includes phenols and biphenols guaiacyl structures (*ortho*-methoxyphenol) variously substituted in the *para* position to the phenolic OH group with electron withdrawing (EWG) or electron donating groups (EDG). Among these compounds, **isoEu** showed the highest antioxidant activity, while **Cr** and **Eu** were less efficient and reactive with similar comparable PFmax and IDmax values. **Va** and **Apo** monomers exhibited eight-fold lower PFmax and IDmax values compared to **isoEu**. These results can be rationalized by the high reactivity of the **isoEu** phenoxy radical stabilized by the presence of a conjugated allylic chain in *para* position of the phenolic OH group. It has been found that **DCr** exhibited a two-fold greater antioxidant efficiency (PF) than that of the corresponding monomer **Cr** even when ID values were comparable. All monomers are structurally similar, differing only by the nature of the *para* side chain. The high antioxidant activity of **isoEu**, **Cr**, and **Eu** compared to **Va** and **Apo** monomers, confirmed the importance of the presence of an electron donating group (EDG) in the *para* position with respect to phenolic OH of the guaiacyl moiety.

Comparison of A2 compounds

**Curc >> M3 > M1 ≥ FA** (**Curcumin** is the strongest antioxidant)

The A2 group is characterized by various α,β-unsaturated chains at the *para* position to the *ortho*-methoxyphenol OH group. Curcumin exhibits a PF 2.5-fold higher than that of **M3** and four-fold higher than that of **M1** and **FA**. Besides, curcumin also shows ID values three-fold higher than that of **M3**, five-fold higher than that of **M1** and seven-fold higher than that of **FA**.

Comparison of A3 compounds

**M5** ≥ **M8** = **M9** > **M4** ≥ **M2** > **HFA**

The A3 group includes phenols and biphenols with a guaiacyl unit variously substituted with saturated chains, that prevents the delocalization of the aryloxy radical so that the substituents do not significantly influence the antioxidant potential. The guaiacyl moiety is the main source of the antioxidant activity and consequently the values of ID and PF were comparable, ranging between 5–8.8 and 3.5–5 values respectively for the monomers **M2, M4, M5, M8** and **M9**, while ID and PF values of the corresponding dimers varied between 5.9–9.8 and 4.9–6.9, respectively.

The PF of **M5** is higher than that of **HFA** (three-fold) and almost the same as for **M2, M4, M8** and **M9** (1.2–1.3-fold). ID of **M5** is higher than that of **HFA** (three-fold) and equal to those of **M8** and **M9** and almost the same as for **M4** and **M2** (1.3- and 1.6-fold), respectively.

It could be concluded that for all monomers:

**Strong antioxidants exhibit PF** ≥ **5 and ID** ≥ **5**

**PF**: **Curc** (13.8) > **isoEu** (10.1) > **M3** (5.8) > **M5** (5.0)

**ID: Curc** (29.3) >> **M3** (9.8) > **M5** (8.8) = **M8** (8.8) = **M9** (8.8) > **isoEu** (6.9) = **M4** (6.8) > **M1** (6.3) > **M2** (5.5) > **HFA** (5.0)

**Moderate antioxidants exhibit 5** > **PF** ≥ **2 and 5** > **ID** ≥ **2**

**PF: M8** (4.3) = **M9** (4.2) > **M4** (3.8) = **Cr** (3.6) = **M1** (3.5) = **M2** (3.5) > **FA** (3.2) = **HFA** (3.2) = **Eu** (3.2) 

**ID: FA** (4.4) > **Cr** (3.5) > **Eu** (2.9)

**Weak activity refers to PF** < **2 and ID** < **2**

**PF: Va** (1.3) = **Apo** (1.3); **ID < 2 Va** (1.3) > **Apo** (0.9)

For the dimers, the following new orders were obtained:

**PFmax: Curcumin** (13.8) = **D1**(13.5) > **D3** (12.8) >> **DCr** (8.8) > **D9** (6.9) > **D8** (5.8) = **D2** (5.8) > **D5** (4.9) > **DHFA** (3.3) = **DFA** (3.3)

**IDmax: Curc** (29.3) = **D1** (29.3) >> **D3** (11.0) > **D9** (9.8) > **D2** (8.8) > **DCr** (7.2) > **D5** (6.3) > **D8** (5.9) > **DHFA** (4.8) ≥ **DFA** (4.2)

Complex estimation of antioxidant properties for all compared *ortho*-methoxyphenols (monomers and dimers):

**Strong antioxidants**: **PFmax** ⩾ **5**; **IDmax** ≥ **5**

**PFmax: Curc** (13.8) = **D1** (13.5) > **D3** (12.8) > **isoEu** (10.1) > **DCr** (7.2) > **D9** (6.9) > **D4** (6.4) > **D8** (5.8) = **D2** (5.8) > **D5** (4.9)

**IDmax: Curc** (29.3) = **D1** (29.3) >> **D3** (11.0) ≥ **D9** (9.8) = **M3** (9.8) > **D2** (8.8) = **M8** (8.8) = **M9** (8.8) = **M5** (8.8) > **D4** (8.0) > **isoEu** (6.9) = **M4** (6.8) > **M1** (6.3) = **D5** (6.3) > **D8** (5.9) ≥ **M2** (5.5) ≥ **HFA** (5.0) ≥ **DHFA** (4.8)

**Moderate antioxidants**: **5 > PFmax** ≥ **2**; **5** > **IDmax** ≥ **2**

**PFmax: M8** (4.3) ≥ **M9** (4.2) ≥ **DHFA** (3.8) > **Cr** (3.6) = **M1** (3.5) = **M2** (3.5) ≥ **DFA** (3.3) = **Eu** (3.2) = **HFA** (3.2) = **FA** (3.0)

**IDmax: FA** (4.4) ≥ **DFA** (4.2) > **Cr** (3.5) > **DCr** (3.2) > **Eu** (2.9)

**Weak antioxidants**: **PFmax** < **2, IDmax** > **2**

**PFmax**: **Va** = **Apo** (1.3)

**IDmax**: **Va** (1.3) > **Apo** (0.9)

⮚ ***Effect of concentration***

**Figure 2:**
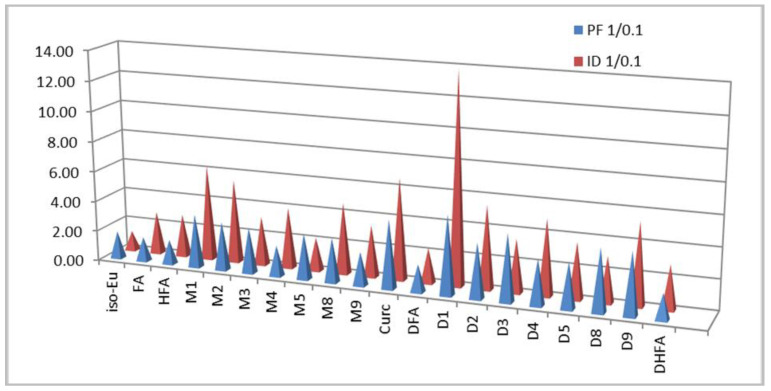
Effect of concentration for selected monomers and dimers.

Figure 2 exhibits a pictorial comparison of the concentration effect assessed for the selected monomers and dimers.

Table S2 with the relevant numerical data is presented in the Supporting Information.

From the results obtained the following conclusion could be made:

Strong concentration effect is manifested by the following characteristics:

**PF_10_ > 4: D1** (5.2) > **Curc** (4.5) = **D3** (4.4) ≥ **D9** (4.1)

**ID_10_ > 4: D1** (13.9) > **Curc** (6.7) = **M1** (6.3) > **M2** = **D2** (5.5) = **D9** (5.4) ≥ **D4** (5.0) ≥ **M8** (4.7) ≥ **M4** (4.0)

Moderate concentration effect is characterized as follows:

**4 > PF_10_ > 2: D2** (3.6) = **M1** (3.5) = **CA** = **HCA** (3.5) ≥ **M2** (3.2) = **M3** (3.0) = **D4** (3.0) = **M5** = **D5** = **M8** (2.9) ≥ **M9** (2.2)

The remaining compounds exhibited a weak effect, with PF_10_ and ID_10_ lower than 2.

⮚ ***Effect of biphenyl structure***

**Figure 3:**
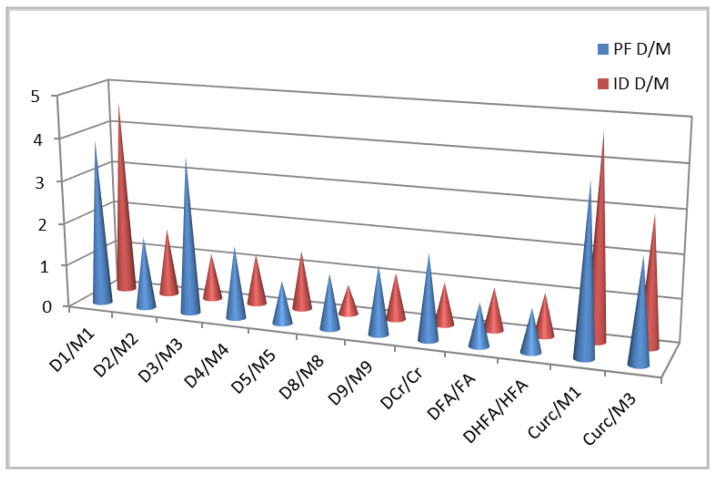
Effect of biphenyl structure. The main kinetic parameters evaluated for dimer/monomer pairs.

The data relevant to examining the effect of biphenyl structure are disclosed in Table S3, presented in the Supporting Information. In a pictorial way the pertinent results are displayed in Figure 3 for selected monomers and dimers.

Dimer **D1**, an α,β-unsaturated ketone, exhibited the highest antioxidant activity comparable to the well-known antioxidant curcumin (**Curc**). **D1** shares many structural features with curcumin and can be considered as a biphenyl analog of **Curc**. Comparison of **D1** with the other compounds showed: (a) four-fold higher PF and five-fold higher ID compared to its monomer **M1**; (b) two-fold higher PF and three-fold higher ID compared to **M3**; (c) similar PF and three-fold higher ID compared to **D3**; (d) four-fold higher PF and seven-fold higher ID compared to **FA** and **DFA** compounds. Concerning the different antioxidant activity between dimers and monomers, the couple **D1/M1** exhibited the biggest differences in antioxidant efficiency (PF_D_/PF_M_ = 4) and antioxidant activity (ID_D_/ID_M_ = 5). **DFA** and **FA** showed similar behavior in both antioxidant properties but were lower than that of the other compounds of the A2 group. Although the unsaturated side chain in position 4 in compounds **FA** and **DFA** should stabilize the phenoxyl radical by resonance, the presence of the COOH group at the end of the side chain is able to induce lipid oxidation through the hydroperoxide decomposition into free radicals [13]. In the couple **D3/M3**, the difference of antioxidant reactivity was negligible meanwhile the antioxidant efficiency of dimer **D3** was two-fold higher than that of its **M3** monomer.

The **DHFA/HFA** couple had ID and PF values reasonably lower than those of the other compounds of the group.

⮚ ***Reaction mechanism for monophenolic antioxidants***

***Ortho-methoxyphenols from A1 group***

**Scheme 1:**
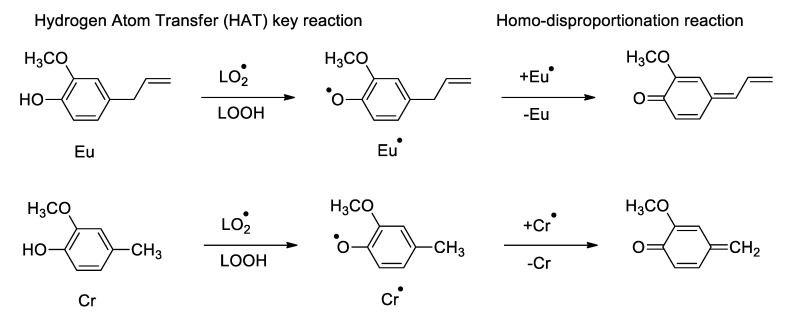
Reaction mechanism for **Eu** and **Cr**.

When comparing **isoEu** with other A1 group studied monomers at low and high concentrations, it is seen that **isoEu** is the monomer with the highest PF activity: 2-fold greater than **Cr,** 2.3-fold greater than **Eu** and 5-fold greater than **Apo** and **Va** at low concentration. However, at high concentration, the effect of **isoEu** increases to 2.8-fold higher compared to **Cr**, 3.2-fold to **Eu** and 7.8 to **Va** and **Apo**, respectively. To explain the obtained results, it must be taken into account that those phenolic antioxidants with labile H atom in *ortho*- or *para*- position towards their phenolic group, are able to be included in disproportionation reaction of their phenoxyl radicals [30]. This reaction is very important for the mechanism of lipid oxidation in the presence of monomeric phenolic antioxidants, because the initial molecule can be regenerated in this reaction and their antioxidant potential increases significantly. Monomeric phenolic antioxidants from group A1, **Eu** and **Cr**, are able to participate in the homo-disproportionation reaction with regeneration of the initial antioxidant molecule (see Scheme 1).

It is important to note that **isoEu**, which differs from **Eu** only in the location of the double bond in the side chain, demonstrates significantly higher antioxidant activity in both concentrations. This difference results in a two-fold higher antioxidant efficiency of **isoEu** at low concentration (0.1 mM) and a three-fold increase at high concentration (1.0 mM), with the stabilization factor (PF) of **isoEu** increasing as much, accordingly. This difference is due to the fact that in **isoEu** the double bond in the side chain leads to elongation of the conjugated system, which in turn provides a greater opportunity to stabilize the radical.

***Ortho-methoxyphenols from A2 group***

**Scheme 2:**
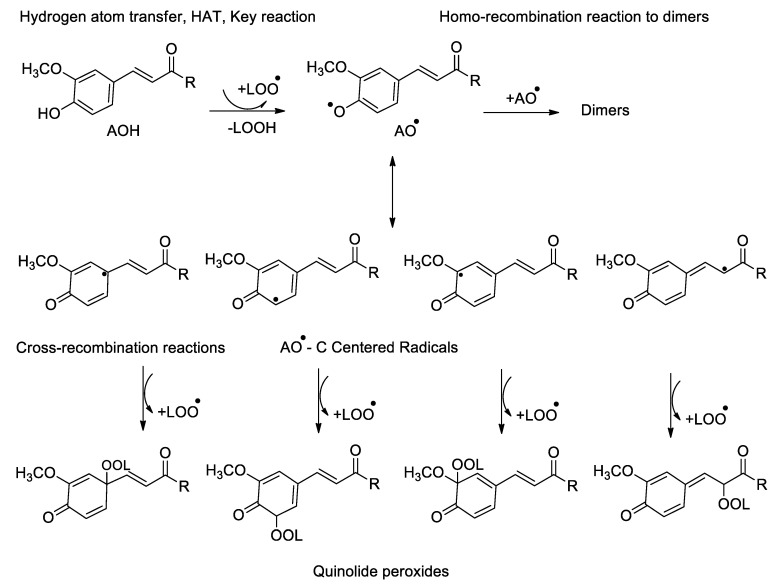
Reaction mechanism for *ortho*-methoxyphenols (group A2) during lipid autoxidation reaction with lipid peroxide radicals, reaction of homo-recombination of phenoxyl radical (AO^•^), resonance structures of AO^•^ (O- and C-centered radicals) and quinolide peroxides formation.

The reaction mechanism for *ortho*-methoxyphenols from A2 group are presented in Scheme 2.

***Ortho-methoxyphenols from A3 group***

**Scheme 3:**
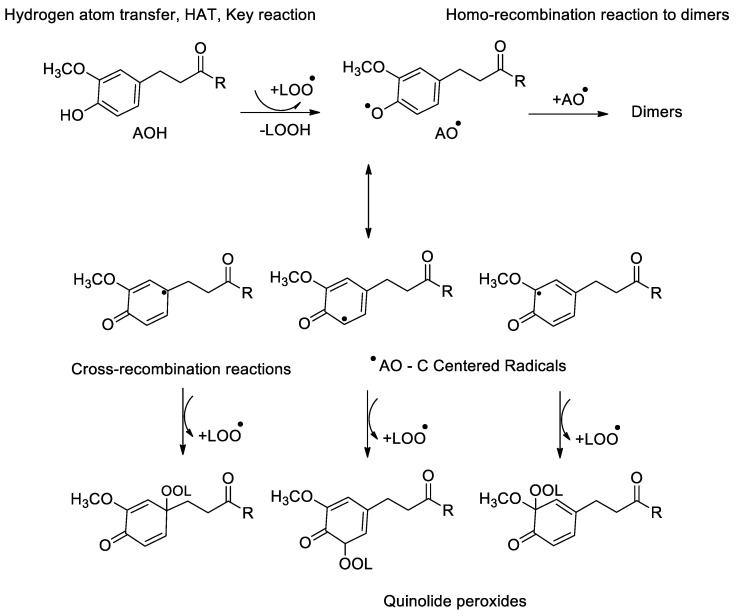
Reaction mechanism for *ortho*-methoxyphenols (group A3) during lipid autoxidation: reactions with lipid peroxide radicals, reactions of homo-recombination of phenoxyl radical (AO^•^), resonance structures of AO^•^ (O- and C-centered radicals) and quinolide peroxide formation.

Scheme 3 depicts the pertinent reaction mechanism for *ortho*-methoxyphenols from group A3.

⮚ ***Activity of ortho-hydroxyphenols (monomers and dimers)***

The experimental results on examining the activity of *ortho*-hydroxyphenols are collected in Table 3.

**New orders of antioxidant activity as manifested by the PF and ID values:**

**0.1 mM PF: HPh** (14.6) > **CA** (8.8) ≥ **HCh** (8.2) > **HCA** (6.7)

**1.0 mM PFmax: HPh** (46.2) > **CA** (33.1) > **HCA** (20.7)

**0.1 mM ID: HPh** (43.8) >> **HCh** (15.2) ≥ **CA** (14.7) > **HCA** (13.7)

**1.0 mM IDmax: HPh** (58.7) >> **HCA** (36.7) > **CA** (28.0)

It could be concluded that **HPh** demonstrates the strongest antioxidant potential in both concentrations, two- to three-fold greater than that of **CA**, **HCh**, and **HCA**. Comparison of catechol structure effect vs. guaiacol reveals the following sequences:

**0.1 mM PF**: **HPh/M1** (12.7) > **HCA/HFA** (5.2) > **CA/FA** (4.5) ⩾ **HCh/Eu** (4.0)

**0.1 mM ID: HPh/M1** (40) > > **HCA/HFA** (10.5) > **CA/FA** (5.8) > **HCh/Eu** (4.8)

**1.0 mM PFmax: HPh/M1** (13.2) > **CA/FA** (10.3) > **HCA/HFA** (6.5)

**1.0 mM IDmax: HPh/M1** (9.3) > **HCA/HFA** (7.3) > **CA/FA** (6.3)

Comparison of B1-B2-B3 compounds

B1–B3 groups include four 3,4-dihydroxy catechols with various substituents at 1 position. All the components of these groups showed stronger antioxidant activities compared with all A1-A3 monomers. The high antioxidant activity of *ortho*-biphenols is due to the presence of the two active OH groups in 3 and 4 positions, this structural feature has the ability to form an intramolecular hydrogen bonding increasing the stability of the phenoxyl radical. Compound **HPh** showed the strongest antioxidant activity (PF = 46.2 and ID = 58.7) evidencing a high reactivity of the phenoxyl radicals due to an extra stabilization on the α,β-unsaturated ketone chain in *para* position to one of the two phenolic OH groups. The relative low PF value of **HCh** could be explained by the presence of an allylic chain that prevents delocalization of the peroxyl radical. Despite of the presence of a terminal COOH group that could enhance lipid oxidation, the **CA** and **HCA** monomer, showed five-fold higher ID and PF values with respect to the corresponding *ortho*-methoxy **FA** and **HFA** compounds.

⮚ ***Reaction mechanism of ortho-hydroxyphenols***

**Scheme 4:**
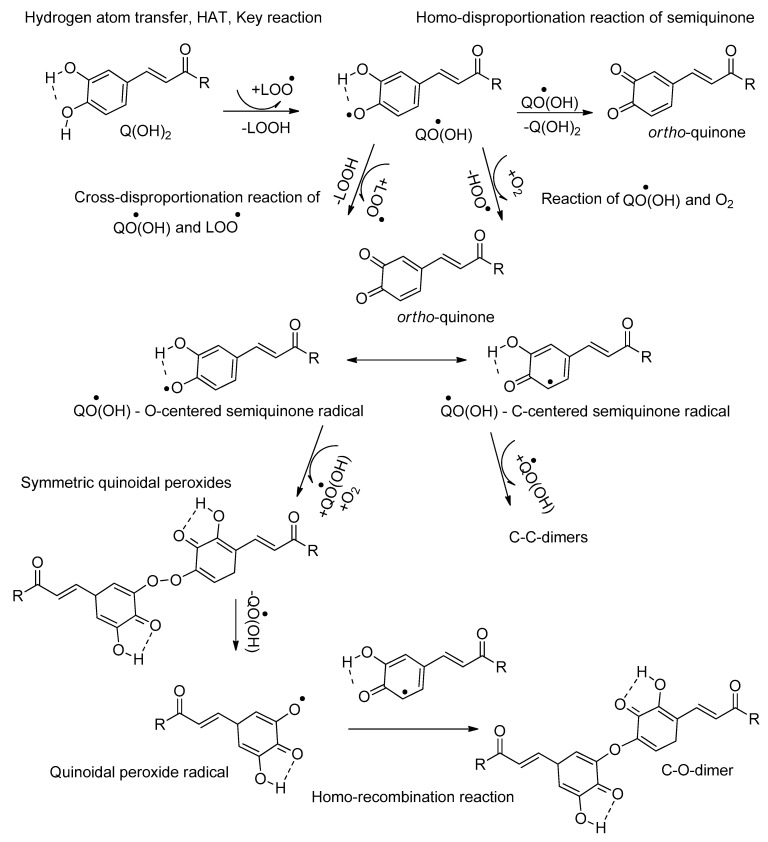
Reaction mechanism for *ortho-hydroxyphenols.*

Scheme 4 depicts the reaction mechanism for *ortho*-hydroxyphenols.

The key reaction of *ortho*-hydroxyphenols is in contrast with that of *ortho*-methoxyphenols. The stoichiometric coefficient of *ortho*-quinones from which semiquinone radicals are formed is n = 2 only in those cases where a semiquinone radical is not involved in other reactions [9]. Semiquinone radicals possess a high reactivity towards oxygen and are able to generate the reactive radicals HO_2_^•^. All tested *ortho*-hydroxyphenols are able to regenerate the initial molecule of antioxidant during the homo-dissproportionation reaction with regeneration of the initial molecule of *ortho*-hydroxyphenols (Scheme 4). Both symmetric and non-symmetric quinoidal peroxides are unstable, and even at low temperature decompose.

Comparison of dimers and monomers

It has been found that **D1** showed stronger antioxidant efficiencies and inhibition degrees than the corresponding monomers **M1** at both concentration 0.1 mM and 1.0 mM. The four-fold higher PF and five-fold higher ID of **D1** dimer is due to the possible formation of various intermediates that are reactive towards LO_2_^•^ radicals. These data are in accordance with our previous work where we reported that, theoretically, one molecule of **D1** is able to trap nine lipid peroxide radicals LO_2_^•^, while monomer **M1** is able to trap only two radicals [18]. In the case of curcumin, which can be regarded as a **M1** dimer, the relative antioxidant kinetic parameters were almost the same as for the couple **D1/M1**. The couples **D1/M1** and **Curc/M1** were the strongest antioxidant compounds due to the presence of an α,β-unsaturated chain at position 1, which can stabilize the phenoxy radicals formed. The couples **D4/M4**, **D5/M5**, **D8/M8**, **D9/M9**, **DFA/FA**, **DHCh/HCh** showed similar PFs and IDs at both concentrations of 0.1 mM and 1.0 mM. Dimers **D3** and **DCr** exhibited higher antioxidant efficiency than the corresponding monomers **M3** and **Cr**, and inhibition degrees were comparable.

⮚ ***Effect of catechol/guaiacol structures of the examined antioxidants***

With respect to the effect of catechol vs. guaiacol structure, we have obtained the following PF and ID trends:

**0.1 mM PF**: **HPh/M1** (14.6) > **HCA/HFA** (5.2) > **CA/FA** (4.5) > **HCh/Eu** (3.3)

**0.1 mM ID**: **HPh/M1** (43.8) >> **HCA/HFA** (10.5) > **CA/FA** (5.8) > **HCh/Eu** (4.8)

**1.0 mM PFmax: HPh/M1** (13.2) > **CA/FA** (10.3) > **HCA/HFA** (6.5)

**1.0 mM IDmax: HPh/M1** (9.3) >> **HCA/HFA** (7.3) > **CA/FA** (6.3)

The results confirm that the presence of a catechol moiety in all cases leads to a higher antioxidant potential. **HPh** demonstrated the highest effect (13- and 44-fold higher activity than that of **M1**). **CA** had a higher antioxidant efficiency (10-fold in terms of PFmax and 6-fold in terms of IDmax) than that of **FA**. **HCA** exhibited a 10- and 7- fold higher antioxidant potential than **HFA**. The ID max of **HCh** is five-fold higher than that of **Eu**.

#### 3.2.2. Chain-Breaking Antioxidant Activity during Initiated Oxidation

**Figure 4:**
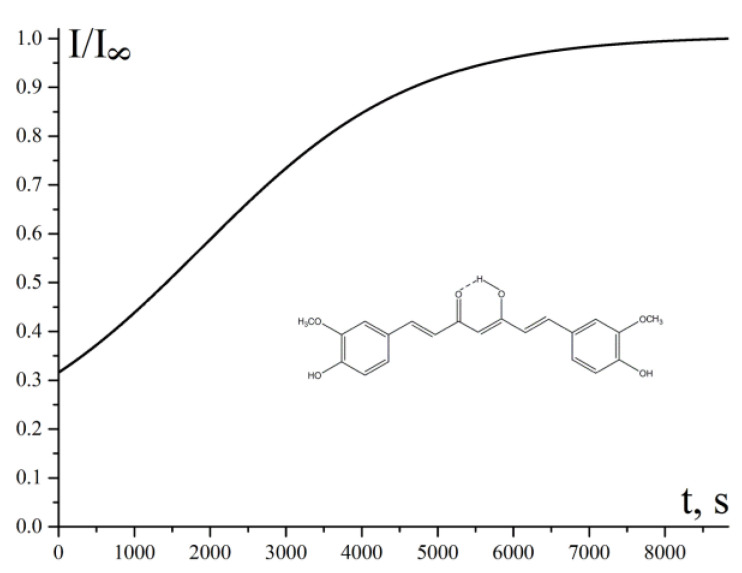
Time profiles of the relative CL intensity (I/I_∞_) measured during the oxidation of ethylbenzene in the aerated chlorobenzene solution (26% vol.) initiated by AIBN at the rate of R_IN_ = 5.1 × 10^–9^ M s^−1^ and 50 °C upon introduction of 1.2 × 10^−5^ M of curcumin.

Figure 4 displays an example of the time profile of the CL intensity upon introduction of curcumin. Processing these experimental data affords the value *k*_A_ = (4.3 ± 0.3) × 10^4^ M^−1^s^−1^. The *k*_A_ values for the other compounds assessed through the same procedure and under the same conditions are displayed in Table 4.

As it is evident from Table 4, the *k*_A_ values for all the antioxidants considered in this work are of the same order, however, the data for the dimers are about 1.5-2.5 times higher than those for the corresponding monomers.

### 3.3. Computational Studies

#### 3.3.1. DFT Calculations

**Figure 5:**
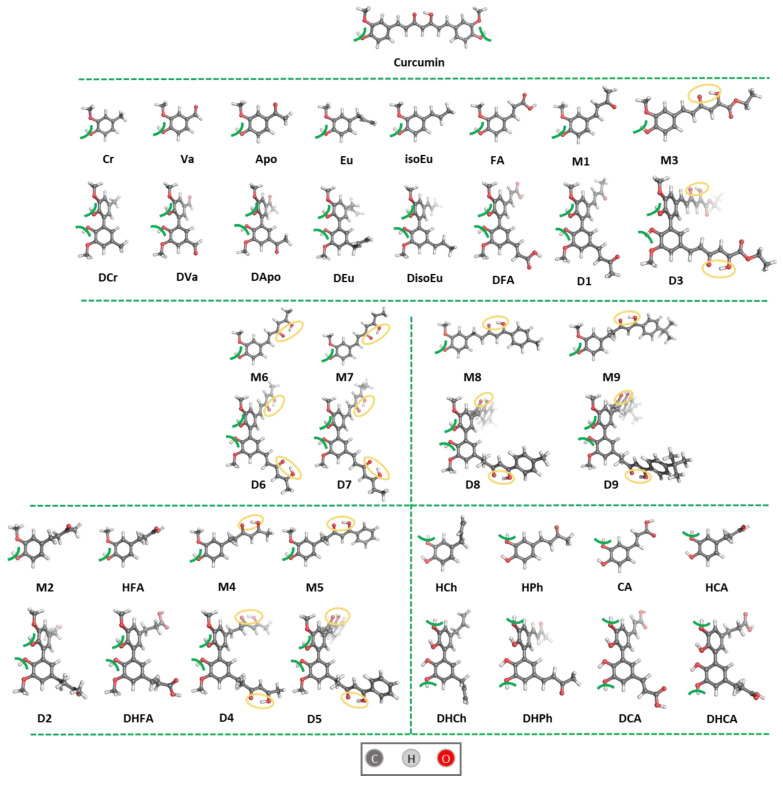
Optimized geometry of the most stable conformers. (Green curved line) OH groups whose BDEs are reported in Table S4 and presented in Figure 6; (yellow orange circle) structural fragment where protopropic tautomerism can occur.

Density functional theory (DFT) has been employed to study the structure and radical scavenging activity of natural bio-antioxidants and their synthetic analogs by computing bond dissociation enthalpy for the O–H chemical bond. The optimized geometries of curcumin (enol form), monomers and dimers in the gas phase are given in Figure 5. Rotamers with intramolecular H-bond between the hydroxy and methoxy/second hydroxy group are considered for curcumin and monomers. Possible rotations around single C-C bonds are taken into account for the R-substituent (monomers), and BDEs are calculated for the most stable ones. From these energetically preferred monomers the respective dimers (hydroxylated biphelyls) are constructed. Biphenyls without H-bond between both rings are considered as they have been found to be preferred over the H-bonded ones in a previous study [13]. The intramolecular H-bond patterns were not confirmed by FT-IR spectroscopy, but according to a recent study of a similar biphenyl system (magnolol derivatives) by Amorati et al. [51], theoretically (DFT) predicted H-bonding pattern correlates with experimentally obtained IR results and reactivity of polyphenols. C2-symmetrical dimers in *syn*-like conformation with dihedral angles θ = 62–64° between the benzene carbon atoms bearing phenolic OH-groups are considered in the study herein for all compounds. It has been demonstrated in a previous study that the differences in the stability between dimers with *syn*-like and *anti*-like conformations are negligible [13]. For compounds **M3–M9** *keto-enol* tautomeric forms presented in Figure 5 are considered. Compound **D7** (not synthesized) is modelled by analogy to the other dimers.

**Figure 6:**
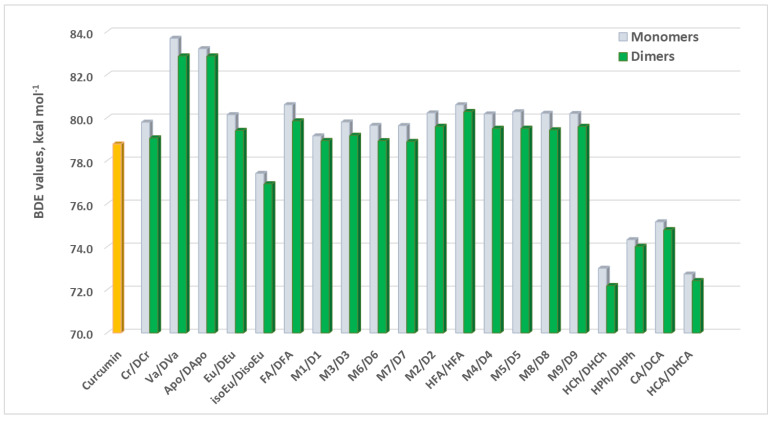
DFT calculated BDEs for curcumin, natural and synthetic antioxidants.

DFT calculated parameters characterizing the parent neutral molecules (AO) and monoradical species (AO^•^) are given in Table S4, SI. The BDEs derived from the respective H_298_ values are presented graphically in Figure 6 and the values are listed in Table S4. BDEs for biradical species generation are not presented—they are always higher than those for the first H-atom abstraction [31].

**Curcumin:** characterized by BDE value of 78.8 kcal mol^−1^, a relatively high value.

**Monomers Cr**, **Va**, **Apo**, **Eu**, **iso-Eu**, **FA**, **M1**, **M3**: characterized by values from 77.4 kcal mol^−1^ to 83.7 kcal mol^−1^. The value calculated for isoEu (77.4 kcal mol^−1^) is lower than that of curcumin (78.8 kcal mol^−1^).

**Monomers HFA**, **M2**, **M4**–**M7**: BDEs from 79.6 kcal mol^−1^ to 80.6 kcal mol^−1^ (the range of the values is 1 kcal mol^−1^). **M6** and **M7** do not differ. Compounds with saturated alkyl chain (**M2**, **HFA**, **M4** and **M5**) also have similar BDEs; from 80.2 to 80.6 kcal mol^−1^. The high BDEs imply an inability of these compounds to generate/stabilize AO^•^ radical species.

**Monomers M8**, **M9**, **HCh**, **HPh**, **CA**, **HCA**: the BDEs for **M8** and **M9** (with saturated alkyl chain) are high. The BDEs for compounds **HCh**, **HPh**, **CA**, **HCA** are low (72.7–75.2 kcal mol^−1^) thanks to the presence of a catechol moiety.

**Dimers DCr**, **DVa**, **DApo**, **DEu**, **DisoEu**, **DFA**, **D1**, **D3**: characterized by BDE values from 79.2 kcal mol^−1^ to 82.9 kcal mol^−1^ (the range is 1.2 kcal mol^−1^). The lowest value is found for DisoEu (79.4 kcal mol^−1^).

**Dimers DHFA**, **D2**, **D4**–**D7**: for this group BDEs are between 78.9 kcal mol^−1^ and 80.3 kcal mol^−1^. **D6** and **D7** do not differ. Compounds with saturated alkyl chain (**D2**, **DHFA**, **D4** and **D5**) are characterized by similar BDEs—from 79.5.2 to 80.3 kcal mol^−1^.

**Dimers D8**, **D9**, **DHCh**, **DHPh**, **DCA**, **DHCA**: high BDEs for **D8** and **D9** (with saturated alkyl chain) and very low values for the rest of compounds (with two catechol moieties), between 72.2 kcal mol^−1^ and 74.8 kcal mol^−1^.

Not surprisingly, the BDEs for compounds (monomers and dimers) with a catechol moiety (**HCh/DHCh**, **HPh/DHPh**, **CA/DCA**, **HCA/DHCA**) are lower than those for compounds with a guaiacol moiety (**curcumin**, **Cr/DCr**, **Va/DVa**, **Apo/DApo**, **Eu/DEu**, **isoEu/DisoEu**, **FA/DFa**, **HFA/DHFA**, **M1**–**M9**/**D1**–**D9**. In the catechol derivatives there is at least one non-engaged in intramolecular H-bond OH group, while in the guaiacol derivatives the OH group is engaged in an H-bond with the adjacent *ortho*-methoxy group. The type of substituent R has a lower impact on the BDE values, suggesting that it is possible to make further modifications to obtain derivatives with desired properties.

Regarding the comparison of monomers with dimers, based on the BDE data reported in Table S4 and presented in Figure 6, it can be seen that BDEs for dimers are lower than those for corresponding monomers, i.e., the biphenyl structure is beneficial for the radical scavenging activity of such compounds.

#### 3.3.2. In Silico Predictions of ADME/Tox Properties

Various physico-chemical/structural parameters like lipophilicity, hydrogen bonding, molecule size, presence of particular structural features, etc., were analyzed in silico for each of the investigated compounds to estimate the behavior of the molecules in a living organism and most prominently, their transport, metabolic stability, affinity to proteins, toxicity. The main output of this analysis is presented in the Table 5, while all in silico predictions are summarized in the Supplementary Data.

To estimate the drug-likeness properties of the investigated compounds, the Lipinski rule of five was applied. Traditionally, this model has been used to aid development of orally bioavailable drugs by providing a reliable initial estimation of the ability of a molecule to cross membrane barriers in the living organism and to reach the site of action. Application of the Lipinski rule of five showed that almost all compounds have good/moderate drug-like properties. Exceptions are **D3** and **D9**, which have two violations per structure (MW, HA, and respectively MW, logP) of the rule.

The analysis of the obtained HIA results outlines the compounds **FA**, **HFA**, **CA**, and **HCA** as potentially having high HIA, while the rest of the compounds are classified as follows: **M2** and **Cr**—moderate-to-high HIA; compounds **M1**, **HPh**, **Apo**, **M4**, **Va** and **Eu** have poor-to-moderate HIA and the rest are predicted to have poor HIA. It should be noted that the above analysis does not include 7 out of 40 structures that have low reliability of the HIA estimation (compounds **DFA**, **D5**, **DHFA**, **DCA**, **DHCA**, **D8** and **D9**) since they are outside the applicability domain of the applied PAMPA QSAR model.

Concerning the evaluation of the transport across BBB the prediction outlined all dimers except the synthetic curcumin analog **D1** to have limited CNS access. In addition, such limited access is predicted for the pairs **FA/DFA**, **HFA/DHFA**, **CA/DCA** and **HCA/DHCA**. The compounds with predicted potential to permeate CNS are all monomers **M1**–**M9**, **Cr**, **isoEu**, as well as the pairs **HPh/DHPh**, **Eu/DEu** and **HCh/DHCh**.

Particular attention in the in silico predictions is paid on the prediction of P-glycoprotein (P-gp) affinity, due to its significant role in the transport of *xenobiotic* compounds as well as its involvement in cancer multidrug resistance. The results showed that the only compound with significant probability to act as a P-gp inhibitor is curcumin. For the rest of the compounds the P-gp inhibitor/substrate probability is low or the reliability index of the prediction is below 0.5, meaning the predictions are outside the applicability domain of the model. An interesting observation is that the newly synthesized bioantioxidants **D1**, **D3**, **D5**–**D9**, although outside the applicability domain of the model, are predicted with high probability (>0.5) to inhibit P-gp. This result suggests the need to check the P-gp inhibition of these compounds in experimental models.

A model to predict inhibitors/substrates of cytochrome P450 (ACD/Percepta software platform) was further applied to investigate the probability that the compounds could be inhibitors/substrates of one of the five major drug metabolizing enzymes—CYP3A4, CYP2D6, CYP2C9, CYP2C19, and CYP1A2. Only **Eu** was predicted as a substrate of CYP1A2. Most of the compounds, however, are outside the applicability domain of the model.

Further, the in silico analysis included the prediction of possible toxic effects of the investigated bioantioxidants. As a first step, the Derek Nexus knowledge-based expert system was applied for the prediction of systemic toxicity in mammals. As already mentioned, in the present study the minimal likelihood to consider a toxic outcome was “plausible”. Eugenol (**Eu**) was predicted to be hepatotoxic with level of likelihood “probable”, suggesting the presence of at least one strong argument that the proposition is true and no arguments against it [49]. The prediction is based on the presence of the hepatotoxicity alert “*para*-alkylphenol or derivative” and experimental evidence in the mouse. The following compounds were predicted as hepatotoxic with a “plausible” level of likelihood based on the presence of the same alert: **M2**, **D2**, **M4**, **D4**, **M5**, **D5**, **HFA**, **DHFA**, **Cr** and **DCr**. Among them, two compounds have also an additional hepatotoxicity alert: “2-arylacetic or 3-arylpropionic acid”—compounds **HFA** and **DHFA**. In addition, carcinogenicity as potential toxicity endpoint was outlined in few cases. Thus, the compounds **CA**, **HPh**, **HCA**, **HCh** were predicted as potentially carcinogenic with level of likelihood “plausible” based on the presence of carcinogenicity alert “catechols with no more than 15 non-hydrogen atoms”.

As a second step, probabilistic categorization was performed using ACD/Percepta toxicity models. Concerning the mutagenicity model (based on Ames test data) many of the investigated compounds were found in the training set of the model and not one of them was mutagenic (compounds **Curc**, **FA**, **M5**, **HFA**, **Va**, **Eu**, **CA** and **HCh**). The rest have a low probability of mutagenicity or are outside the applicability domain of the model. The results on the newly synthesized bioantioxidants **D1**, **D6** and **DHPh** deserves particular attention, since they demonstrate high probability for mutagenicity in the model (>0.5), although being outside its applicability domain. Thus, studying these compounds with the Ames test would be particularly worthwhile.

The cardiotoxicity predictions were based on the hERG inhibition model in ACD/Percepta. A low probability of cardiotoxicity is demonstrated for **D6** (0.26). The rest of the compounds show even lower probability and/or are outside the applicability domain of the model.

The compounds’ endocrine disruption potentials were estimated by prediction of ERα-binding in the ACD/Percepta platform. The analysis showed that none of the compounds demonstrated a significant likelihood of binding ERα.

Concerning acute toxicity endpoint, the predictions were generated as a list of probabilities that the compound’s LD_50_ (rat, oral route) would exceed the cut-off values which separate different hazard categories as defined by OECD guidelines [52]. According to the predictions, none of the investigated compounds is assigned to hazard category I–III (LD_50_ < 300 mg/kg).

### 3.4. Comparison between Experimentally Obtained and Theoretically Predicted Antioxidant and Biological Activity

The experimental and computational DFT results obtained for the studied compounds are in line. DFT methods provide some insights into the molecular structure and intrinsic properties of the studied natural and natural-like antioxidants (monomers and dimers). BDE values for the dimers with biphenyl structure are slightly lower than the values, obtained for the corresponding monomers. This fact is in agreement with the experimental findings. In silico predictions of ADME/Tox properties of the investigated compounds gives an estimate of their probable behavior in a living organism and particularly their transport, metabolic stability, affinity to proteins, toxicity, etc.

The antioxidant compounds used in this comparative study belong to the class of hydroxylated/methoxylated phenyls and biphenyls widely distributed in nature. Hydroxylated biphenyls, present in many naturally occurring compounds of high biologic importance, such as ellagitannins and vancomicyn, play a fundamental role in biosystems due to their unique pharmacophore structure comprising two aromatic rings bridged by a single C-C bond. Many bioactive curcumin analogs based on hydroxylated biphenyl and phenyl skeleton have been synthesized previously. In particular, we observed high anti-misfolding activity and ligand capacity with alpha-synuclein by dehydrozingerone, zingerone and relative dimers [32], high antiproliferative and apoptotic activity against malignant melanoma by C2-symmetrycal hydroxylated biphenyls bearing a β,β-unsaturated ketone chain [35], high activity against clinical fusarium strains by prenylated *trans*-cinnamic esters and ethers [53], fungicidal activity and micotoxyn production inhibition against fusarium culmorum fungal plant pathogen by phenyl propanoids and a dimer of cinnamic acids [54] and antioxidant activity in bulk oils on lipid autoxidation by zingerone and dehydrozingerone derivatives [13].

In silico predicted possible toxic effects of the investigated antioxidants, especially of those with a catechol structure (**CA**, **HPh**, **HCA**, **HCh**), gave the warning that compounds with catechol moiety are carcinogens. No information is available on the carcinogenic effects of catechols in humans, but there is animal carcinogenicity data available in the literature [55,56]. Several plant phenols (including caffeic acid) have been demonstrated to exert carcinogenic potential in rats [57].

## 4. Conclusions

A set of 40 natural and natural-like phenolic and biphenolic compounds, nine of which are newly synthesized, were selected for a comparative study of their chain-breaking antioxidant activities and thus as modulators of oxidative stress. This included natural and natural-like *ortho*-methoxy and *ortho*-hydroxy phenols and biphenols, most of them being curcumin-like compounds. The presence of a catechol structure and/or an α,β-unsaturated ketone chain seems to be of key importance for efficient chain-breaking antioxidant activity, probably due to a resonance stabilization of the generated phenoxyl radical. The **HPh** catechol, bearing an α,β-unsaturated chain in the *para* position, showed the highest antioxidant activity both in terms of protection factor (PF) and degree of inhibition (ID). Generally, dimers had higher activity than the corresponding monomers. The drug safety profiles of the investigated structures were explored in silico. The predicted possible toxic effects of the compounds with catechol structure emphasize the importance of modifying the structure in order to improve the desired properties and to reduce the unwanted ones.

## 5. Outlook

The experimental and theoretical results presented herein could motivate a deeper study on the synthesis of new hydroxylated natural and natural-like phenols and biphenols, with new functionalities in key positions of the aromatic ring in order to obtain new natural-like compounds with improved antioxidant and ADME/Tox properties.

## Figures and Tables

**Table 1:** Structures of all studied antioxidants.

**(A) *Ortho*-methoxyphenols**		
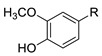	R	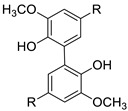
(A1)		
**Cr**	–CH_3_	**DCr**
**Va**	–CHO	**DVa**
**Apo**	–COCH_3_	**DApo**
**Eu**	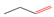	**DEu**
**IsoEu**	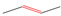	**DisoEu**
(A2)		
**FA**	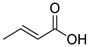	**DFA**
**M1**	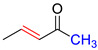	**D1**
**M6**	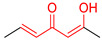	**D6**
**M7**	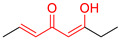	**-**
**M3**	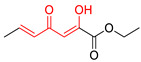	**D3**
**Curc**	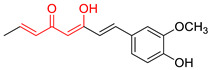	-
(A3)		
**HFA**	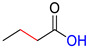	**DHFA**
**M2**	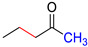	**D2**
**M4**	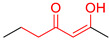	**D4**
**M5**	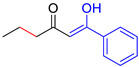	**D5**
**M8**	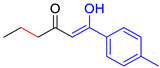	**D8**
**M9**	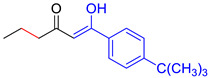	**D9**
**(B) *Ortho*-hydroxyphenols**		
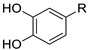	R	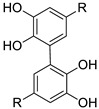
(B1)		
**HCh**	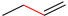	**DHCh**
(B2)		
**HPh**	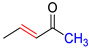	**DHPh**
**CA**	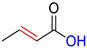	**DCA**
(B3)		
**HCA**	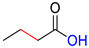	**DHCA**

Non-systematic and systematic names of the studied compounds are given in Table S1, Supporting Information.

**Table 2:** Main kinetic parameters of chain-breaking antioxidant activity of the studied *ortho*-methoxyphenols (monomers and dimers) during lipid autoxidation.

Abbr.	PF 0.1 mM	PF 1.0 mM	Effect	ID 0.1 mM	ID 1.0 mM	Effect	References
Monomers							
(A1)							
**Cr**	2.7	3.6	Moderate	3.3	3.5	Moderate	[31]
**Va**	1.1	1.3	Weak	1.3	1.3	Weak	[31]
**Apo**	1.1	1.3	Weak	0.7	0.9	Weak	[31]
**Eu**	2.5	3.2	Moderate	2.4	2.9	Weak	[31]
***iso*-Eu**	5.6	10.1	Strongest	5.2	6.9	Strong	[31]
(A2)							
**FA**	1.5	3.2	Moderate	1.6	4.4	Moderate	[13,24]
**M1**	1.0	3.5	Moderate	1.0	6.3	Strong	[13]
**M3**	1.9	5.8	Strong	3.1	9.8	Strongest	[13]
**Curc**	3.1	13.8	Strongest	4.4	29.3	Strongest	[13]
(A3)							
**HFA**	1.3	3.2	Moderate	1.3	5.0	Moderate	Tw
**M2**	1.1	3.5	Moderate	1.0	5.5	Strong	[13]
**M4**	1.9	3.8	Moderate	1.5	6.8	Strong	Tw
**M5**	1.7	5.0	Strong	4.0	8,8	Strong	[25]
**M8**	1.5	4.3	Moderate	2.7	8.8	Strong	[25]
**M9**	1.9	4.2	Moderate	2.6	8.8	Strong	[25]
Dimers							
**DCr**	5.2	7.2	Strong	2.1	3.2	Moderate	[31]
**DFA**	1.8	3.3	Moderate	1.8	4.2	Moderate	[13]
**D1**	2.6	13.5	Strongest	2.1	29.3	Strongest	[13]
**D3**	2.9	12.8	Strongest	3.1	11.0	Strongest	[13]
**DHFA**	1.6	3.8	Moderate	1.6	4.8	Moderate	Tw
**D2**	1.6	5.8	Strong	1.6	8.8	Strong	[13]
**D4**	2.1	6.4	Strong	1.7	8.0	Strong	Tw
**D5**	1.6	4.9	Moderate	1.7	6.3	Strong	[25]
**D8**	1.4	5.8	Strong	2.0	5.9	Strong	[25]
**D9**	1.7	6.9	Strong	1.8	9.8	Strongest	[25]

Tw—this work; **DVa**, **DApo**, **DEu** are not soluble in lipid substrate, **M6**, **M7**, **D6**, **D7**, **DisoEu** are studied only theoretically.

**Table 3:** The main kinetic parameters of the chain-breaking activity of *ortho*-hydroxyphenols and their dimers during lipid autoxidation.

Abbr.	PF 0.1 mM	PF 1.0 mM	Activity	ID 0.1 mM	ID 1.0 mM	Activity	Refs.
**CA**	6.7	33.1	Strongest	9.3	28.0	Strongest	[23]
**HPh**	14.6	46.2	Strongest	43.8	58.7	Strongest	tw
**HCh**	8.2	15.2	Strongest	n.d.	n.d.	-	tw
**HCA**	6.7	20.7	Strongest	13.7	36.7	Strongest	tw
**DHCh**	12.5	15.2	Strongest	n.d.	n.d.	-	tw
**DCA**	n.s.	n.s.	-	n.s.	n.s.	-	tw
**DHPh**	n.s.	n.s.	-	n.s.	n.s.	-	tw
**DHCA**	n.s.	n.s.	-	n.s.	n.s.	-	tw

n.s. refers to not soluble in lipid substrate, n.d. means not determined, tw pertains to this work.

**Table 4:** The values of the effective rate constant (*k*_A_) of scavenging the peroxyl radicals of ethylbenzene by antioxidants.

Abbr.	Compound	*k*_A_ (M^−1^s^−1^)
**Curc**	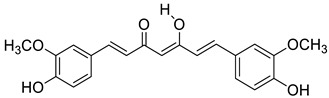	(4.3 ± 0.3) × 10^4^
**FA**	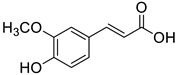	(1.6 ± 0.1) × 10^4^
**DFA**	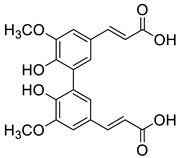	(4.1 ± 0.3) × 10^4^
**M1**	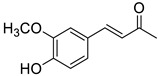	(1.7 ± 0.1) × 10^4^
**D1**	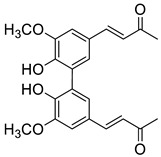	(4.2 ± 0.3) × 10^4^
**M2**	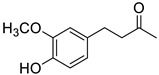	(2.6 ± 0.3) × 10^4^
**D2**	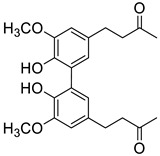	(5.1 ± 0.3) × 10^4^
**M3**	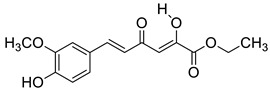	(2.4 ± 0.3) × 10^4^
**D3**	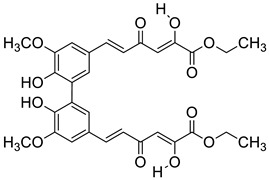	(3.7 ± 0.3) × 10^4^

**Table 5:** In silico predictions of important ADME/Tox properties of the investigated compounds.

Compound	logP	logS	MAD (%HIA)	BBB	P-gp	Mammal Hepato-Toxicity	Mammal Carcino-Genicity	Hazard Category
A1								
**Cr**	1.83	−1.70	27.7 (28%)	P	0.05	A		IV
**DCr**	2.57	−3.31	0.3 (0%)	P	0.11 *	A		IV
**Va**	1.29	−1.41	11.5 (11%)	W	0.04			IV
**DVa**	1.53	−2.20	1.0 (1%)	N	0.08			V
**Apo**	1.4	−1.70	13.8 (14%)	P	0.06			IV
**DApo**	1.83	−3.13	0.2 (0%)	N	0.22			IV
**Eu**	2.48	−2.08	11.3 (11%)	P	0.06	**A**		IV
**DEu**	3.43	−4.27	0.0 (0%)	P	0.16 *	A		V
**iso-Eu**	2.55	−2.30	9.3 (9%)	P	0.05	A		IV
**Diso-Eu**	3.60	−4.56	0.0 (0%)	P	0.13	A		V
A2								
**FA**	1.68	0.71	139.9 (100%)	N	0.02			V
**DFA**	1.86	0.41	35.0 (35%) *	N	0.02 *			V
**M1**	1.69	−2.02	14.9 (15%)	P	0.10			IV
**D1**	2.13	−3.97	0.1 (0%)	P	0.57 *			V
**M6**	1.62	−2.35	2.9 (3%)	P	0.09 *			IV
**D6**	2.32	−4.74	0.0 (0%)	W	0.60 *			V
**M7**	1.98	−2.67	1.8 (2%)	P	0.10 *			IV
**M3**	1.03	−2.56	1.2 (1%)	P	0.14 *			IV
**D3**	1.75	−5.01	0.0 (0%)	N	0.70 *			V
**Curc**	2.64	−3.63	0.4 (0%)	P	0.40			V
A3								
**HFA**	1.27	0.71	529.1 (100%)	N	0.03	A, B		V
**DHFA**	1.17	0.41	34.3 (34%) *	N	0.02	A, B		V
**M2**	1.1	−1.44	55.6 (56%)	P	0.07	A		V
**D2**	1.06	−2.96	1.9 (2%)	W	0.21 *	A		V
**M4**	1.18	−2.30	11.3 (11%)	P	0.08	A		IV
**D4**	1.83	−4.80	0.2 (0%)	W	0.39 *	A		V
**M5**	2.41	−3.81	3.7 (4%)	P	0.19 *	A		IV
**D5**	4.16	−7.26	0.0 (0%) *	N	0.76 *	A		IV
**M8**	2.86	−3.89	1.4 (1%)	P	0.22 *	A		IV
**D8**	4.76	−7.69	0.0 (0%) *	N	0.84 *	A		IV
**M9**	3.83	−4.64	0.4 (0%)	P	0.42 *	A		V
**D9**	7.2	-8.95	0.0 (0%) *	N	0.94 *	A		V
B1								
**HCh**	1.9	−1.00	9.7 (10%)	P	0.04	A	C	IV
**DHCh**	2.92	−2.85	0.1 (0%)	P	0.06	A		IV
B2								
**HPh**	1.68	−1.03	14.4 (14%)	P	0.04		C	IV
**DHPh**	1.92	−2.86	0.3 (0%)	P	0.12 *			V
**CA**	1.35	0.74	124.7 (100%)	W	0.01		C	V
**DCA**	1.32	0.45	10.8 (11%) *	N	0.00			V
B3								
**HCA**	0.83	0.74	476.3 (100%)	N	0.02	A, B	C	IV
**DHCA**	0.46	0.44	10.7 (11%) *	N	0.01	A, B		V

**Legend:** Lipophilicity (logP); water solubility (logS at pH 7.4); MAD (maximum absorbable dose) and based on it %HIA (human intestinal absorption, % of 100 mg dose); BBB (blood–brain barrier) penetration: P—penetrant, N—non-penetrant, W—weak penetrant; mammal hepatotoxicity: A—“para-alkylphenol or derivative”, B—“2-arylacetic or 3-arylpropionic acid”; mammal carcinogenicity: C—“catechol with no more than 15 non-hydrogen atoms”; hazard category based on probabilistic categorization of acute systemic toxicity (based on LD_50_). *—the compound is outside the applicability domain of the model; bold text—“probable” level of likelihood.

## Data Availability

Not applicable.

## Supplementary Materials

The following are available online at https://www.mdpi.com/article/10.3390/antiox10040624/s1, Figure S1: ^1^H and ^13^C NMR spectra of M4, M6 and M7. Figure S2: ^1^H and ^13^C NMR spectra of D4 and D6. Figure S3: ^1^H and ^13^C NMR spectra of DisoEu, DHCh, DCA and DHCA. Table S1: All the studied compounds summarized with their names according IUPAC nomenclature, trivial names and their abbreviations. Table S2: Effect of the 10-fold higher concentration of the studied antioxidants (0.1 mM and 1.0 mM). Table S3: Effect of biphenyl structure. Table S4: B3LYP/6-31+G(d,p) gas phase calculated enthalpies H_298_ (a.u.) and dihedral angles (°) for AO structures shown in Figure 5 and respective radicals (AO^•^). BDEs (kcal mol^−1^) are derived from H_298_ values calculated for neutral (AO) and radical (AO^•^, H^•^) species. Scheme S1: Basic scheme of lipid (LH) autoxidation. Scheme S2: Basic kinetic scheme of uninhibited and inhibited (in presence of monomers and dimers) lipid autoxidation. Scheme S3: Basic kinetic scheme of initiated oxidation of ethylbenzene (RH) in CL, in absence and in presence of studied compounds; Excel table: In silico predictions of ADME/Tox properties.
