# Supplementary material for: Natural Chain-Breaking Antioxidants and Their Synthetic Analogs as Modulators of Oxidative Stress
Source: Antioxidants (Basel). 2021 Apr 19;10(4):624. doi: 10.3390/antiox10040624 (PMC8074124; doi:10.3390/antiox10040624)
Supplement: Supplementary file 1 [file antioxidants-10-00624-s001.zip › antioxidants-1182777-supplementary.pdf]

Supplementary Material for

Natural chain-breaking antioxidants and their synthetic analogs as modulators of oxidative stress

Vessela D. Kancheva[1*], Maria Antonietta Dettori[2] , Davide Fabbri[2], Petko Alov[3], Silvia E. Angelova[4*], Adriana K.Slavova-Kazakova[1], Paola Carta[2], Valerii A. Menshov[5,] Olga I. Yablonskaya[5], Aleksei V. Trofimov[5,6], Ivanka Tsakovska[3], Luciano Saso[7]

[1] *Institute of Organic Chemistry with Centre of Phytochemistry, Bulgarian Academy of Sciences, "Acad. G. Bonchev" Str., bl.9, Sofia 1113, Bulgaria; vessy.kancheva@abv.bg, adriana.kazakova@orgchm.bas.bg*
[2] *CNR, Istituto di Chimica Biomolecolare, Traversa La Crucca 3, 07100 Sassari, Italy, mariaantonietta.dettori@cnr.it, davidegaetano.fabbri@cnr.it, paola.carta@cnr.it*
[3] *Institute of Biophysics and Biomedical Engineering, Bulgarian Academy of Sciences, "Acad. G. Bonchev" Str., bl.21, 1113 Sofia, Bulgaria; petko@biophys.bas.bg, itsakovska@biomed.bas.bg*
[4] *Institute of Optical Materials and Technologies "Acad. J. Malinovski", Bulgarian Academy of Sciences, "Acad. G. Bonchev" Str., bl.109, 1113 Sofia, Bulgaria, sea@iomt.bas.bg*
[5] *Emanuel Institute of Biochemical Physics, Russian Academy of Sciences, "Kosigina"4 Str., 119334 Moscow, Russian Federation, vinoman66@mail.ru, olga.yablonsky@gmail.com, avt_2003@mail.ru*
[6] *Moscow Institute of Physics and Technology (National Research University), Dolgoprudny, Moscow Region, Institutskiy per. 9, 141701, Russia*
[7] *Department of Physiology and Pharmacology "Vittorio Erspamer", Sapienza University P.le Aldo Moro 5,00185, Rome, Italy; luciano.saso@uniroma1.it*

Correspondence authors: Prof. Vessela D. Kancheva, PhD, (Former family name Kortenska) Phone: +359 988934995; fax: +359 28700225; e-mail: vessy.kancheva@abv.bg; Assoc. Prof. Silvia E. Angelova, PhD, Phone: +359888726508; e-mail: sea@iomt.bas.bg

Contents

1. Nonsystematic and systematic names of the studied compounds. S3

2. Spectral characterization of the newly synthesized compounds: S5

   2.1. Compounds M4, M6 and M7 .... S5

   2.2. Compounds D4 and D6 .... S5

   2.3. Compounds DisoEu, DHCh, DCA and DHCA S5

   2.4. ^{1}H and ^{13}C NMR spectra S7

3. Lipid autoxidation S12

   3.1. Lipid sample S12

   3.2. Lipid autoxidation S13

   3.3. Determination of the main kinetic parameters of the studied compounds S14

4. Estimation of rate constant of antioxidant reaction with peroxyl radicals (k_A) using kinetic chemiluminescence (CL) method S15

5. DFT computational studies S17

References S19

1. **Nonsystematic and systematic names of the studied compounds.**

Table S1. All the studied compounds summarized with their names according IUPAC nomenclature, trivial names and their abbreviations:

Abbr.	Nonsystematic/trivial name	IUPAC systematic name
**FA**	Ferulic Acid	(E)-3-(4-hydroxy-3-methoxyphenyl)prop-2-enoic acid
**DFA**		(2E,2'E)-3,3'-(6,6'-dihydroxy-5,5'-dimethoxy-[1,1'-biphenyl]-3,3'-diyl)diacrylic acid
**CA**	Caffeic Acid	(E)-3-(3,4-dihydroxyphenyl)acrylic acid
**DCA**		(2E,2'E)-3,3'-(5,5',6,6'-tetrahydroxy-[1,1'-biphenyl]-3,3'-diyl)diacrylic acid
**Cr**	Creosol	2-methoxy-4-methylphenol
**DCr**		3,3'-dimethoxy-5,5'-dimethyl-[1,1'-biphenyl]-2,2'-diol
**Va**	Vanillin	4-hydroxy-3-methoxybenzaldehyde
**DVa**		6,6'-dihydroxy-5,5'-dimethoxy-[1,1'-biphenyl]-3,3'-dicarbaldehyde
**Apo**	Apocinin	1-(4-hydroxy-3-methoxyphenyl)ethanone
**DApo**		1,1'-(6,6'-dihydroxy-5,5'-dimethoxy-[1,1'-biphenyl]-3,3'-diyl)diethanone
**Eu**	Eugenol	2-methoxy-4-prop-2-enylphenol
**DEu**		5,5'-diallyl-3,3'-dimethoxy-[1,1'-biphenyl]-2,2'-diol
**isoEu**	iso-Eugenol	2-methoxy-4-[(E)-prop-1-enyl]phenol
**DisoEu**		3,3'-dimethoxy-5,5'-di((E)-prop-1-en-1-yl)-[1,1'-biphenyl]-2,2'
**M1**	Dehydrozingerone	(E)-4-(4-hydroxy-3-methoxyphenyl)but-3-en-2-one
**D1**		(3E,3'E)-4,4'-(6,6'-dihydroxy-5,5'-dimethoxy-[1,1'-biphenyl]-3,3'-diyl)bis(but-3-en-2-one)
**M2**	Zingerone	4-(4-hydroxy-3-methoxyphenyl)butan-2-one
**D2**		4,4'-(6,6'-dihydroxy-5,5'-dimethoxy-[1,1'-biphenyl]-3,3'-diyl)bis(butan-2-one)
**M3**		(2Z,5E)-ethyl 2-hydroxy-6-(4-hydroxy-3-methoxyphenyl)-4-oxohexa-2,5-dienoate
**D3**		(2Z,2'Z,5E,5'E)-diethyl 6,6'-(6,6'-dihydroxy-5,5'-dimethoxy-[1,1'-biphenyl]-3,3'-diyl)bis(2-hydroxy-4-oxohexa-2,5-dienoate)

**M4**		(Z)-5-hydroxy-1-(4-hydroxy-3-methoxyphenyl)hex-4-en-3-one
**D4**		(1E,4Z)-1-(2',6-dihydroxy-5'-((Z)-5-hydroxy-3-oxohex-4-en-1-yl)-3',5-dimethoxy-[1,1'-biphenyl]-3-yl)-5-hydroxyhexa-1,4-dien-3-one
**M5**		(Z)-1-hydroxy-5-(4-hydroxy-3-methoxyphenyl)-1-phenylpent-1-en-3-one
**D5**		(1Z,4E)-5-(2',6-dihydroxy-5'-((Z)-5-hydroxy-3-oxo-5-phenylpent-4-en-1-yl)-3',5-dimethoxy-[1,1'-biphenyl]-3-yl)-1-hydroxy-1-phenylpenta-1,4-dien-3-one
**M6**		(1E,4Z)-5-hydroxy-1-(4-hydroxy-3-methoxyphenyl)hexa-1,4-dien-3-one
**D6**		(1E,1'E,4Z,4'Z)-1,1'-(6,6'-dihydroxy-5,5'-dimethoxy-[1,1'-biphenyl]-3,3'-diyl)bis(5-hydroxyhexa-1,4-dien-3-one)
**M7**		(1E,4Z)-5-hydroxy-1-(4-hydroxy-3-methoxyphenyl)hepta-1,4-dien-3-one
**M8**		(1Z,4E)-1-hydroxy-5-(4-hydroxy-3-methoxyphenyl)-1-(p-tolyl)penta-1,4-dien-3-one
**D8**		(1Z,1'Z)-5,5'-(6,6'-dihydroxy-5,5'-dimethoxy-[1,1'-biphenyl]-3,3'-diyl)bis(1-hydroxy-1-(p-tolyl)pent-1-en-3-one)
**M9**		(1Z,4E)-1-(4-(tert-butyl)phenyl)-1-hydroxy-5-(4-hydroxy-3-methoxyphenyl)penta-1,4-dien-3-one
**D9**		(1Z,1'Z)-5,5'-(6,6'-dihydroxy-5,5'-dimethoxy-[1,1'-biphenyl]-3,3'-diyl)bis(1-(4-(tert-butyl)phenyl)-1-hydroxypent-1-en-3-one)
**HCh**	Chavicol	(E)-4-(prop-1-en-1-yl)benzene-1,2-diol
**DHCh**		5,5'-di((E)-prop-1-en-1-yl)-[1,1'-biphenyl]-2,2',3,3'-tetraol
**HPh**		(E)-4-(3,4-dihydroxyphenyl)but-3-en-2-one
**DHPh**		(3E,3'E)-4,4'-(5,5',6,6'-tetrahydroxy-[1,1'-biphenyl]-3,3'-diyl)bis(but-3-en-2-one)
**HFA**		3-(4-hydroxy-3-methoxyphenyl)propanoic acid
**DHFA**		3,3'-(6,6'-dihydroxy-5,5'-dimethoxy-[1,1'-biphenyl]-3,3'-diyl)dipropanoic acid
**HCA**		3-(3,4-dihydroxyphenyl)propanoic acid
**DHCA**		3,3'-(5,5',6,6'-tetrahydroxy-[1,1'-biphenyl]-3,3'-diyl)dipropanoic acid
**Curc**	Curcumin	(1E,6E)-1,7-bis(4-hydroxy-3-methoxyphenyl)hepta-1,6-diene-3,5-dione

2. **Spectral characterization of the newly synthesized compounds:**

2.1. Compounds M4, M6 and M7

(*Z*)-5-hydroxy-1-(4-hydroxy-3-methoxyphenyl)hex-4-en-3-one **M4**. (85%): mp 38-40°C (lit.[1] 40-41 °C); (β-ketoenol tautomer): ^{1}H-NMR δ 2.03 (s, 3H), 2.55 (t, *J* = 7.7 Hz, 2H), 2.85 (t, *J* = 7.7 Hz, 2H), 3.87 (s, 3H), 5.45 (s, 1H), 6.50 (bs, 1H), 6.64 (dd, *J* = 2.0, 8.2 Hz, Ar, 1H), 6.65 (d, *J* = 2.0 Hz, 1H), 6.80 (d, *J* = 8.2 Hz, 1H); ^{13}C-NMR δ 24.82, 31.26, 41.38, 55.85, 100.01, 110.95, 114.32, 120.79, 132.60, 143.99, 146.41, 191.17, 193.25; Anal. Calcd. for $C_{13}H_{16}O_4$: C, 66.09; H, 6.83; Found: C, 66.13; H, 6.96.

(*Z*)-1-hydroxy-5-(4-hydroxy-3-methoxyphenyl)-1-phenylpent-1-en-3-one **M6**. (64%): mp 142-143°C (lit.[2] 139.8-141.4 °C); (β-ketoenol tautomer): ^{1}H-NMR δ 2.16 (s, 3H), 3.94 (s, 3H), 5.63 (s, 1H), 6.03 (bs, 1H), 6.33 (d, *J*=16 Hz,1H), 6.92 (d, *J*=8.2 Hz,1H), 7.04 (d, *J*=1.9 Hz, 1H), 7.08 (dd, *J*=1.9, 8.2Hz, 1H), 7.55 (d, *J*=16 Hz, 1H); ^{13}C-NMR δ 26.77, 55.91, 100.66, 109.51, 114.82, 120.28, 122.62, 127.63, 140.06, 146.81, 147.52, 177.96, 196.66; Anal. Calcd. for $C_{13}H_{14}O_4$: C, 66.66; H, 6.02; Found: C, 66.63; H, 6.16.

(1*E*,4*Z*)-5-hydroxy-1-(4-hydroxy-3-methoxyphenyl)hepta-1,4-dien-3-one **M7**. (58%): mp 141-143°C; (β-ketoenol tautomer): ^{1}H-NMR δ 1.18 (t, *J*=7.5 Hz, 3H), 2.42 (q, *J*=7.5 Hz, 2H), 3.95 (s, 3H), 5.64 (s, 1H), 5.86 (bs, 1H), 6.34 (d, *J*=16 Hz,1H), 6.92 (d, *J*=8.2 Hz,1H), 7.03 (d, *J*=1.9 Hz, 1H), 7.09 (dd, *J*=1.9 Hz, 8.2Hz, 1H), 7.52 (d, *J*=16 Hz, 1H); ^{13}C-NMR δ 9.44, 33.23, 55.96, 99.56, 109.48, 114.81, 120.50, 122.62, 127.77, 139.73, 146.78, 147.66, 177.70, 200.00; Anal. Calcd. for $C_{14}H_{16}O_4$: C, 67.73; H, 6.50; Found: C, 67.75; H, 6.56.

2.2. Compounds D4 and D6

(1*E*,4*Z*)-1-(2',6-dihydroxy-5'-((Z)-5-hydroxy-3-oxohex-4-en-1-yl)-3',5-dimethoxy-[1,1'-biphenyl]-3-yl)-5-hydroxyhexa-1,4-dien-3-one **D4** (65%): mp 66-67 °C; (β-ketoenol tautomer): ^{1}H-NMR δ 2.03 (s, 6H), 2.59 (t, *J* = 7.2 Hz, 4H), 2.85 (t, *J* = 7.2 Hz, 4H), 3.89 (s, 6H), 5.48 (s, 2H), 6.10 (bs, 2H), 6.64 (s, 2H), 6.73 (s, 2H); ^{13}C-NMR δ 24.82, 31.36, 40.29, 56.09, 100.14, 110.55, 122.81, 124.45, 132.59, 141.01, 147.24, 191.14, 193.23; Anal. Calcd. for $C_{26}H_{30}O_8$: C, 66.37; H, 6.43; Found: C, 66.33; H, 6.45.

(1*E*,1'*E*,4*Z*,4'Z)-1,1'-(6,6'-dihydroxy-5,5'-dimethoxy-[1,1'-biphenyl]-3,3'-diyl)bis(5-hydroxyhexa-1,4-dien-3-one) **D6** (64%): mp 170-171 °C; (β-ketoenol tautomer): ^{1}H-NMR (DMSO d_6) δ 2.09 (s, 6H), 3.87 (s, 6H), 5.82 (s, 2H), 6.63 (d, *J*=15.6 Hz,2H), 7.07 (d, *J*=2 Hz, 2H), 7.30 (d, *J*=2 Hz, 2H), 7.49 (d, *J*=15.6 Hz, 2H), 9.10 (bs, 2H); ^{13}C-NMR δ (DMSO d_6) 26.91, 56.47, 100.99, 110.28, 120.30, 125.23, 125.82, 125.89, 140.73, 147.00, 148.31, 148.36, 178.62; Anal. Calcd. for $C_{26}H_{26}O_8$: C, 66.94; H, 5.62; Found: C, 66.99; H, 6.66.

2.3. Compounds DisoEu, DHCh, DCA and DHCA

*3,3′-dimethoxy-5,5′-di((E)-prop-1-en-1-yl)-[1,1′-biphenyl]-2,2′-**DisoEu***

^{1}H-NMR δ (acetone d_6) 1.85 (dd, *J*=1.6. Hz, 6.8 Hz, 6H), 3.93 (s, 6H), 5.62 (bs, OH), 6.10-6.18 (series of m, 2H), 6.34 (d, *J*=1.6. Hz, 2H), 6.85 (d, *J*=2.0 Hz, 2H), 7.01 (d, *J*=2.0 Hz, 2H); ^{13}C-NMR δ (acetone d_6) 18.38, 56.03, 107.46, 121.56, 122.38, 125.23, 129.36, 131.12, 143.26, 147.84. Anal. Calcd. for $C_{20}H_{22}O_4$ C, 73.60; H, 6.79; Found: C, 73.65; H, 6.83.

5,5′-diallyl-[1,1′-biphenyl]-2,2′,3,3′-tetraol ***DHCh***

^{1}H-NMR δ (acetone d_6) 3.31 (d, *J*=1.6. Hz, 4H), 5.04 (m, 2H), 5.93 (m, 4H), 6.71 (d, *J*=2.0. Hz, 2H), 6.78 (d, *J*=2.0. Hz, 2H), 7.87 (bs, 2 OH), 8.03 (bs, 2 OH), ^{13}C-NMR δ (acetone d_6) 39.41, 114.45, 114.72, 121.72, 126.52, 132.46, 138.15, 139.75, 145.83. Anal. Calcd. for $C_{18}H_{18}O_4$ C, 72.47; H, 6.08; Found: C, 72.51; H, 6.11.

(2E,2′E)-3,3′-(5,5′,6,6′-tetrahydroxy-[1,1′-biphenyl]-3,3′-diyl)diacrylic acid ***DCA***

^{1}H-NMR δ (DMSO d_6) 6.15 (d, *J*=16.0 Hz, 2H), 6.89 (d, *J*=2.0 Hz, 2H), 7.02 (d, *J*=2.0 Hz, 2H), 7.41 (d, *J*=16.0 Hz, 2H); ^{13}C-NMR δ (DMSO d_6) 113.08, 115.66, 123.98, 125.22, 126.32, 145.16, 145.97, 146.25, 168.40. Anal. Calcd. for $C_{18}H_{14}O_8$ C, 60.34; H, 3.94; Found: C, 60.44; H, 3.98

3,3′-(5,5′,6,6′-tetrahydroxy-[1,1′-biphenyl]-3,3′-diyl)dipropanoic acid ***DHCA***

^{1}H NMR δ (MeOH d_4) 2.24 (t, *J*=7.2 Hz, 4H), 2.61 (t, *J*=7.2 Hz, 4H), 6.34 (d, *J*=2.0. Hz, 2H), 6.57 (d, *J*=2.0. Hz, 2H);^{13}C-NMR δ (MeOH d_4) 23.90, 31.97, 110.57, 119.38, 127.34, 128.53, 147.48, 174.46, 216. 65. Anal. Calcd. for $C_{18}H_{18}O_8$ C, C, 59.67; H, 5.01; Found: C, C, 59.71; H, 5.21.

2.4. ^{1}H and ^{13}C NMR spectra

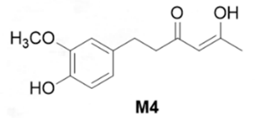

M4

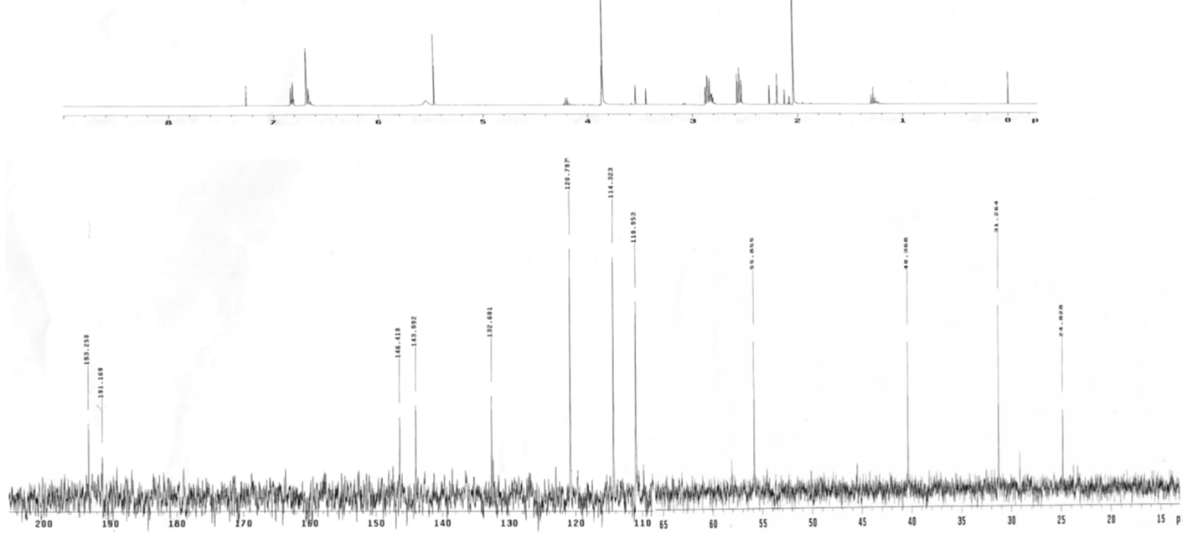

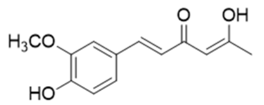

M6

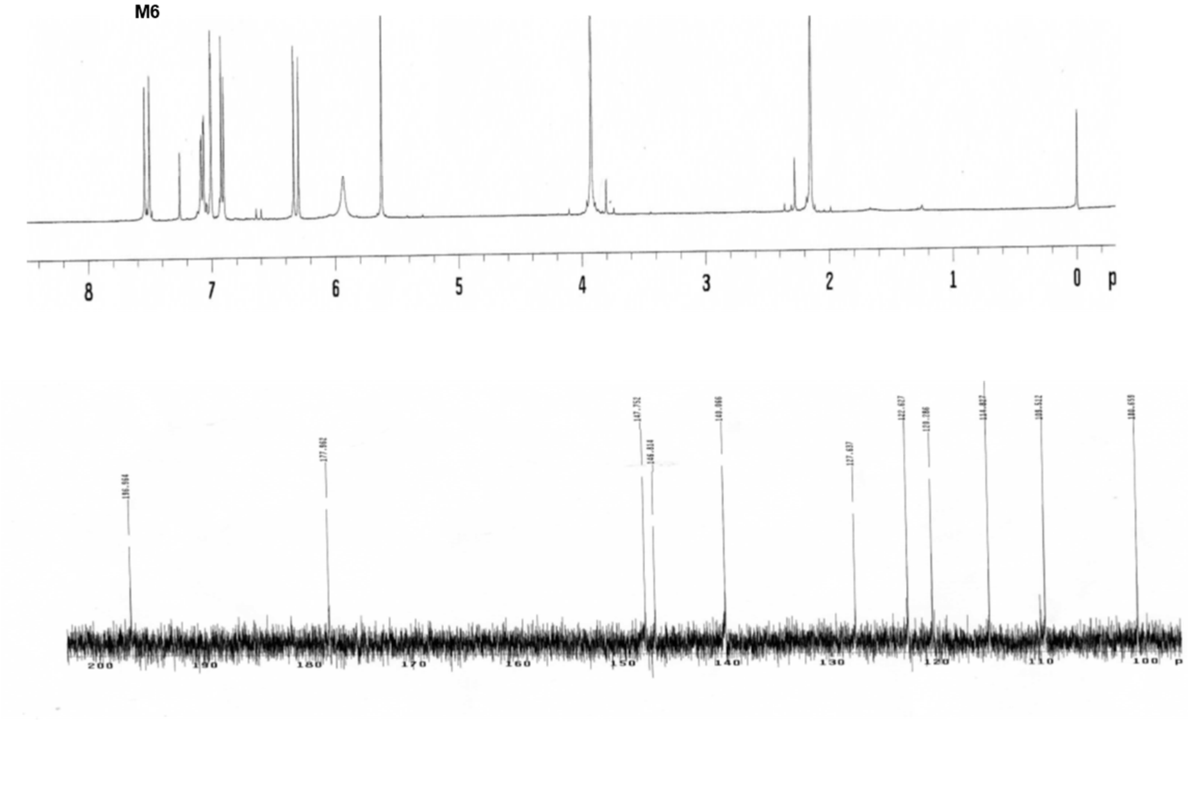

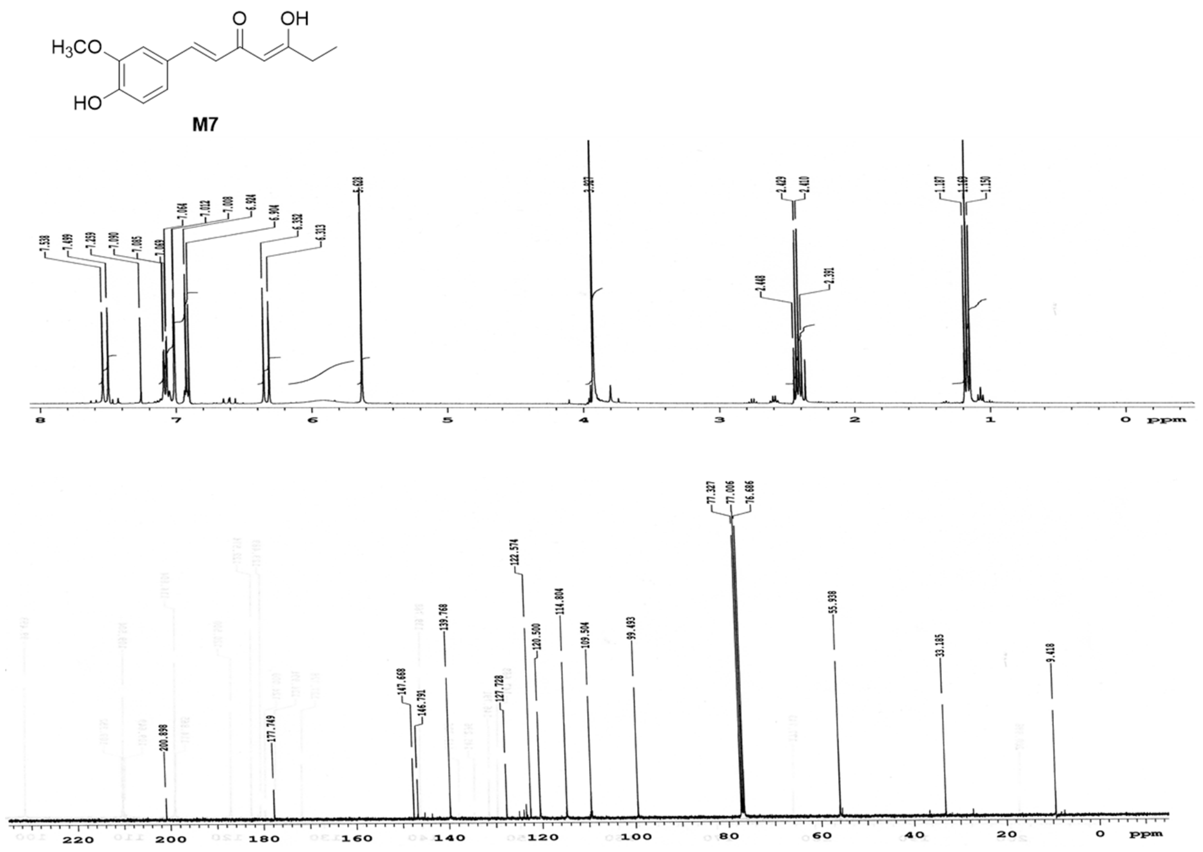

Figure S1. ^{1}H and ^{13}C NMR spectra of **M4**, **M6** and **M7**.

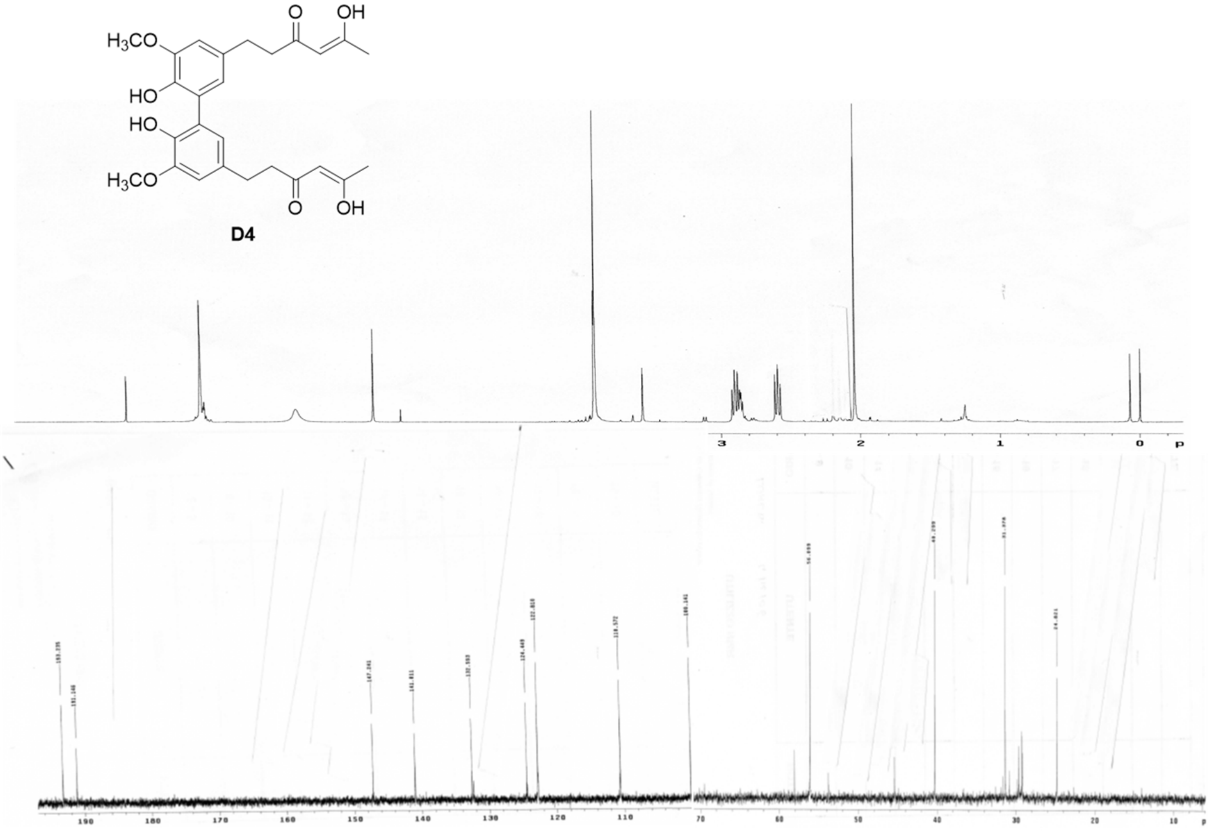

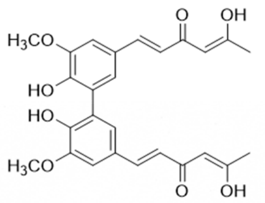

D6

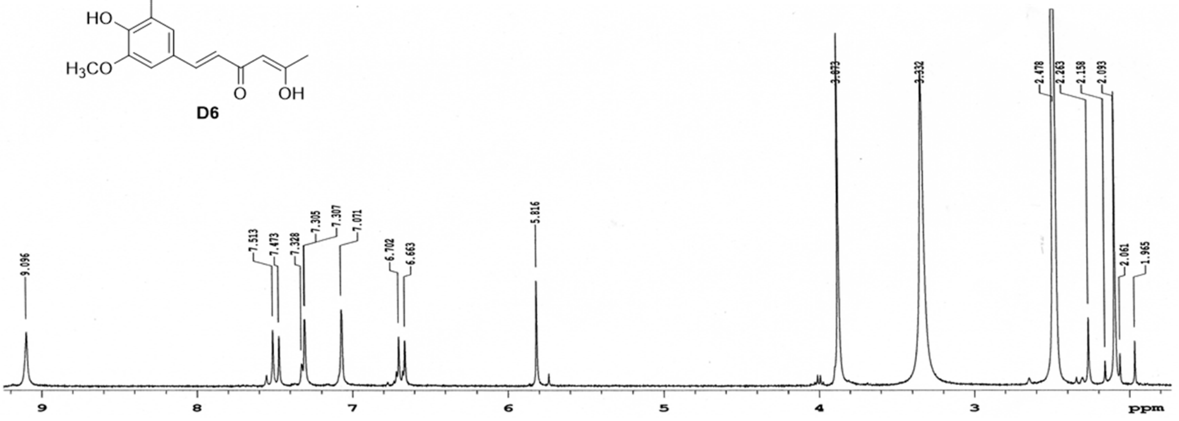

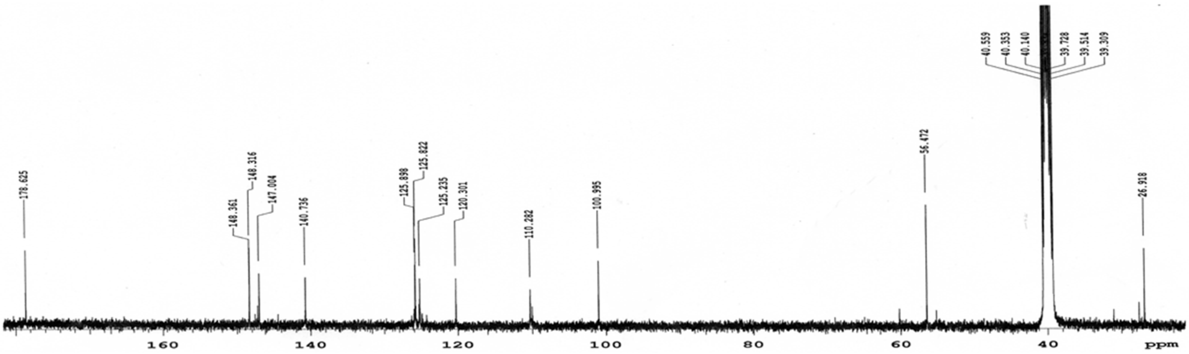

Figure S2. ^{1}H and ^{13}C NMR spectra of **D4** and **D6**.

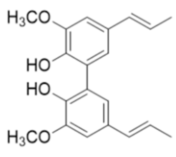

DisoEu

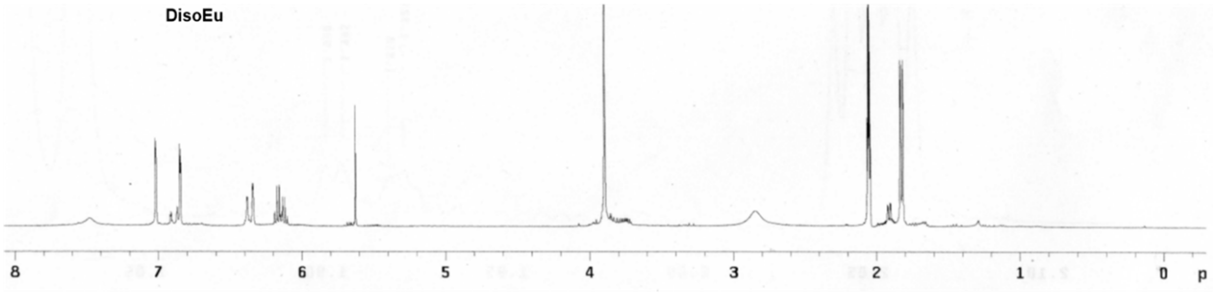

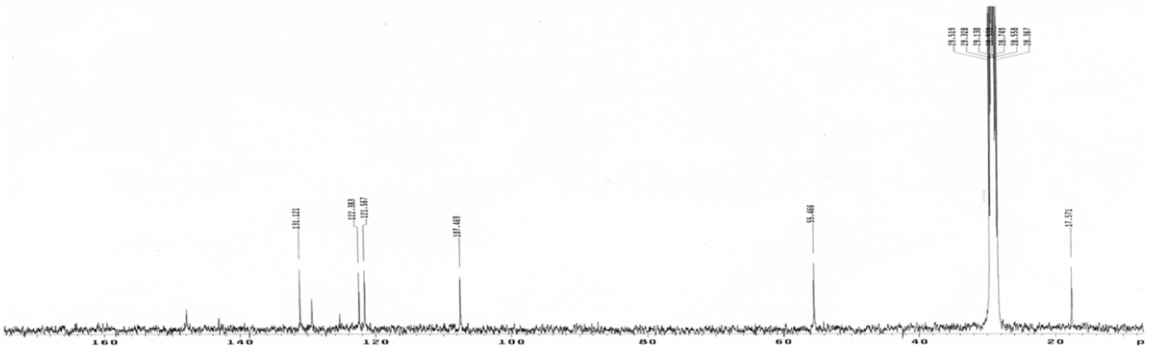

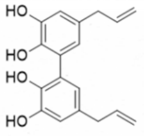

DHCh

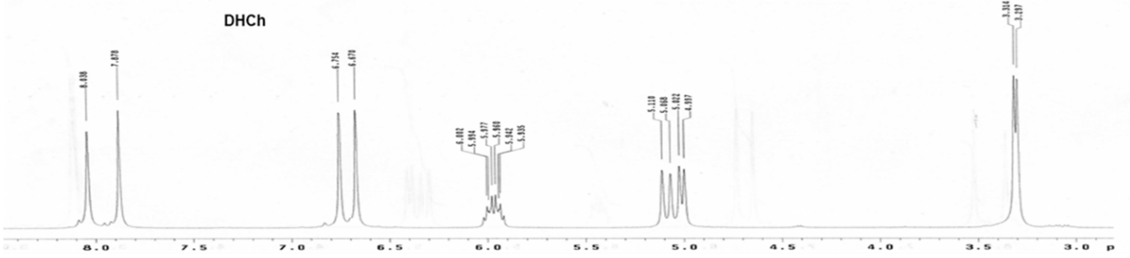

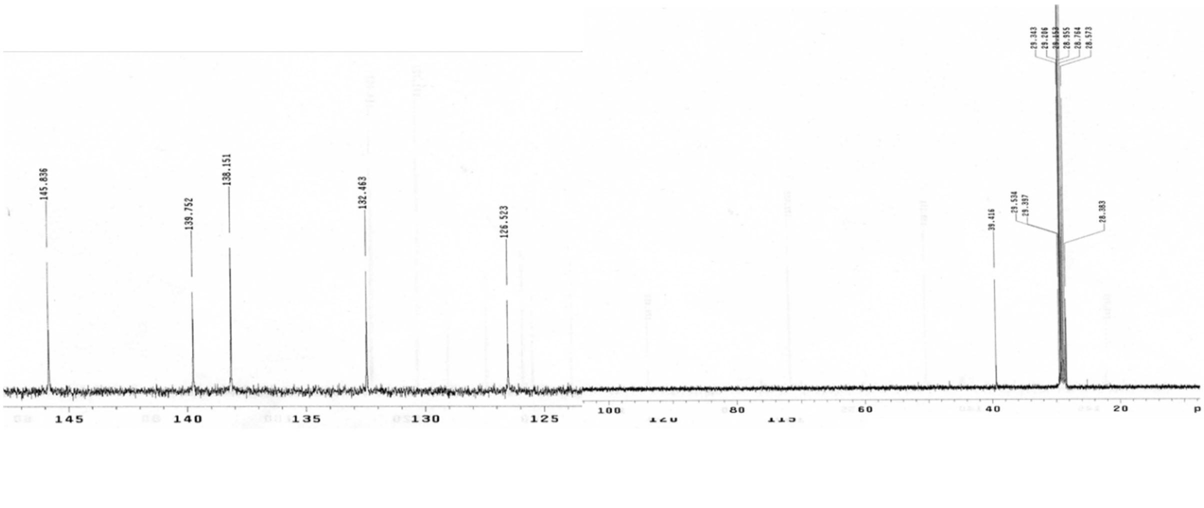

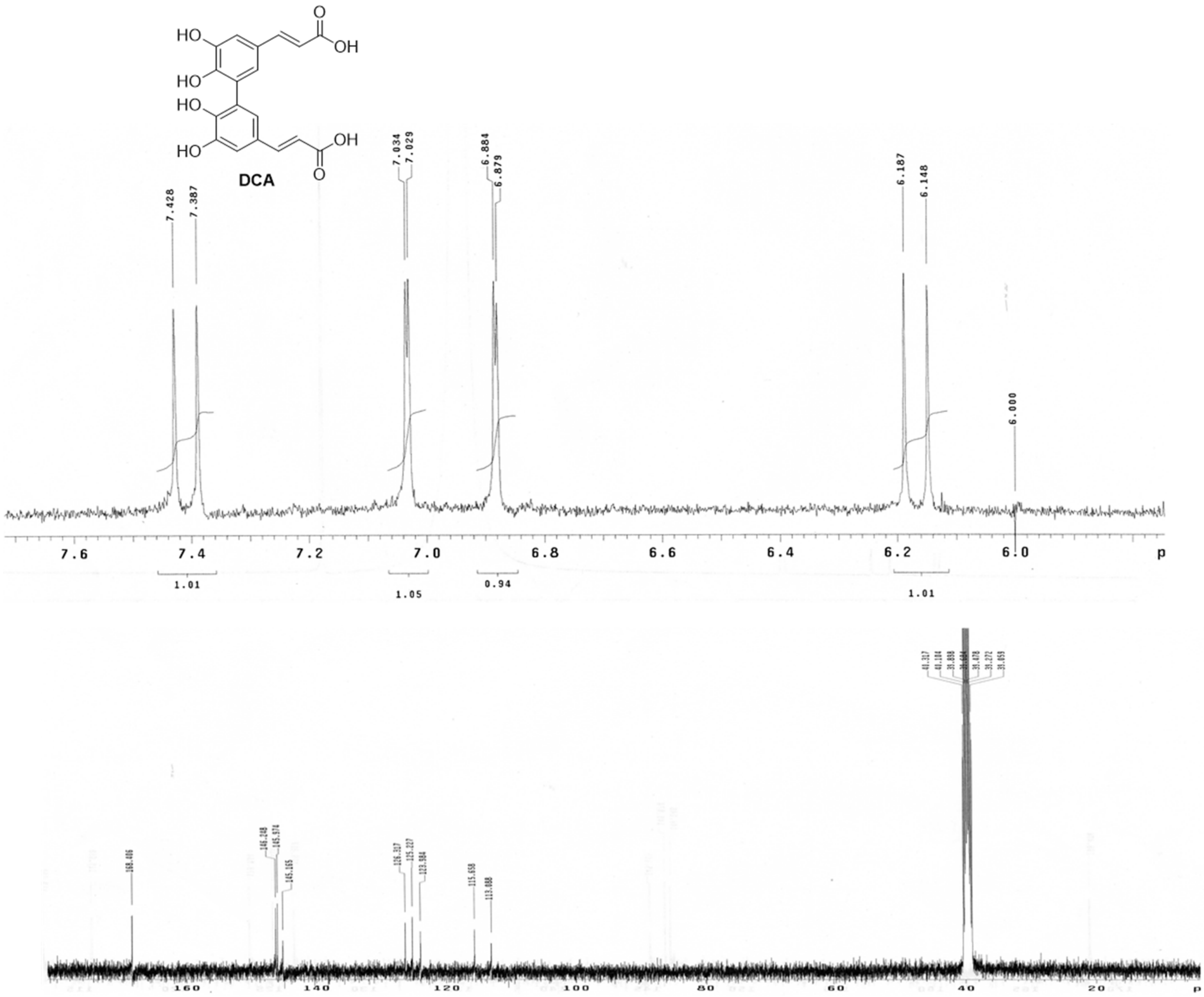
HO
HO
HO
HO
OH
OH
O
O
DCA
7.428
7.387
7.034
7.029
6.884
6.879
6.187
6.148
6.000
7.6
7.4
7.2
7.0
6.8
6.6
6.4
6.2
6.0
p
1.01
1.05
0.94
1.01
168.486
146.248
145.974
145.165
126.317
125.227
123.984
115.658
113.088
160
140
120
100
80
60
40
20
p

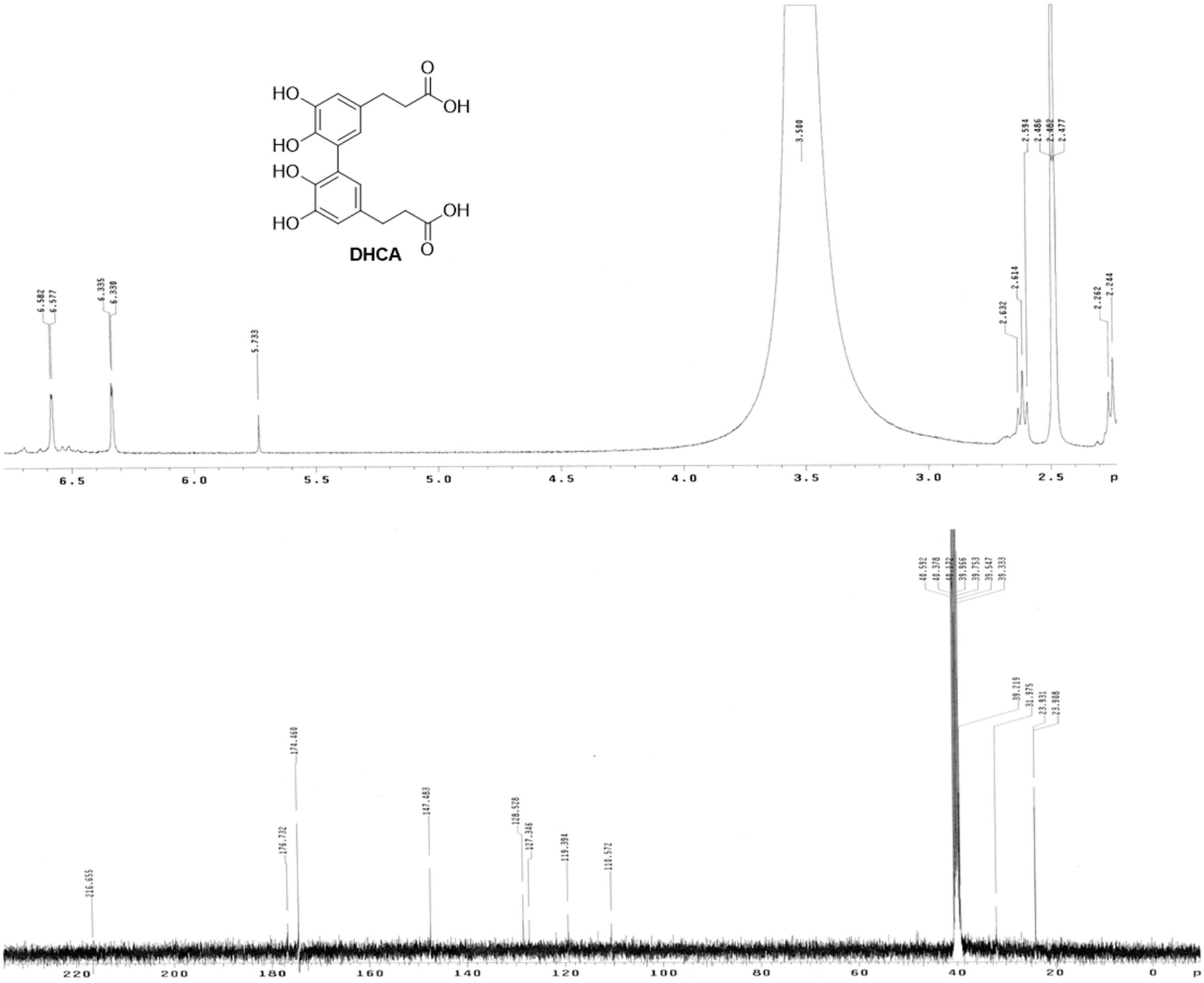

Figure S3. ^{1}H and 13C NMR spectra of **DisoEu**, **DHCh**, **DCA** and **DHCA**.

3. **Lipid autoxidation**

3.1. Lipid sample

Triacylglycerols of commercially available sunflower oil (TGSO) were cleaned from pro- and antioxidants by adsorption chromatography [3] and stored under nitrogen atmosphere at minus 20 °C. Fatty acid composition of the lipid substrate was determined according to Christie [4] by GC analysis of the methyl esters of the total fatty acids obtained with a GC-FID Hewlett-Packard 5890 equipment (Hewlett-Packard GmbH, Austria) and a capillary column HP INNOWAX (polyethylene glycol mobile phase, Agilent Technologies, USA) 30 m × 0.25 mm × 0.25 mm. The temperature gradient started from 165°C increased to 230 °C with 4°C/min and was held at this temperature for 15 min; injection volume was 1 µl. Injector and detector temperatures were 260 and 280 °C, respectively. Nitrogen was the carrier gas at a flow rate of 0.8 ml/min.

Lipid samples containing various inhibitors were prepared directly before use. Aliquots of the antioxidant solutions in acetone were added to the lipid sample. Solvents were removed under a nitrogen flow.

3.2. Lipid autoxidation

The process was carried out in a thermostatic bath at 80 °C (± 0.2 °C) by blowing air through the samples in special vessels and was monitored by withdrawing samples at measured time intervals and subjecting them to iodometric determination of the primary products (lipid hydroxyperoxides, LOOH) concentration, i.e. the peroxide value (PV) [5],[6]. All kinetic data are expressed as the average of two independent measurements.

TGSO is a mixture of different types of fatty acids glycerol esters, but only those fatty acids with pentadiene structures are vulnerable to oxidation by atmospheric oxygen. Phenolic antioxidants inhibit or retard lipid oxidation by interfering with either chain propagation or initiation by readily donating hydrogen atoms to lipid peroxyl radicals.

Scheme S1. Basic scheme of lipid (LH) autoxidation

Non-inhibited lipid (LH) autoxidation (in absence of an antioxidant)		
Chain generation	$2LH + O_2 \rightarrow \delta\ LO_2^{\bullet}$	(R_{IN})
Chain propagation	$LO_2^{\bullet} + LH\ (+O_2) \rightarrow LOOH + LO_2^{\bullet}$	(k_p)
Chain termination	$LO_2^{\bullet} + LO_2^{\bullet} \rightarrow P$	($2k_t$)
Chain branching 1	$LOOH\ (+O_2) \rightarrow \delta_1\ LO_2^{\bullet} + P_1$	(k_{b1})
Chain branching 2	$LOOH + LH\ (+O_2) \rightarrow \delta_2\ LO_2^{\bullet} + P_2$	(k_{b2})
Chain branching 3	$LOOH + LOOH\ (+O_2) \rightarrow \delta_3\ LO_2^{\bullet} + P_3$	(k_{b3})
Inhibiting reactions, responsible to the antioxidant activity of monomers M_iOH		
Chain termination with H atom transfer reaction	$LO_2^{\bullet} + M_iOH \rightarrow LOOH + M_iO^{\bullet}$	(k_{Am})
Chain termination with cross-recombination	$LO_2^{\bullet} + M_iO^{\bullet} \rightarrow$ inactive products	(k'_{Am})
Chain termination with homo-recombination	$2M_iO^{\bullet} \rightarrow$ inactive products	(k_{tMO})
Side reactions of $M_iO^{\bullet}$, decreasing the antioxidant activity of M_iOH		
Reverse inhibition reaction	$M_iO^{\bullet} + LOOH \rightarrow LO_2^{\bullet} + M_iOH$	(k_{-Am})
Additional propagation reaction	$M_iO^{\bullet} + LH\ (+O_2) \rightarrow \delta_3\ LO_2^{\bullet} + M_iOH$	(k_{pMO})
Quinoidal peroxides (QP) formation	$2M_iO^{\bullet} + O_2 \rightarrow QP$	(k_{QP})
Quinoidal peroxides (QP) decomposition	$QP\ (+ LH, + O_2) \rightarrow \delta_4\ LO_2^{\bullet} + P_4$	(k'_{QP})

In this mechanism: LH is linoleic acid with its allylic hydrogen; M_iOH - the studied monomeric *ortho*-metoxyphenol; $LO_2^{\bullet}$ - lipid peroxide radicals; $M_iO^{\bullet}$- phenoxyl radicals; P , P_A, P_1—P_4 – corresponding products formed; QP- quidoinal peroxides; R_{IN} - the rate of initiation; k_P, k_t, k_b, $k_{Am,}$ k'_{Am} , k_{tMO}, k_{pMO}, k_{QP} – corresponding rate constants of different reactions.

In the presence of an antioxidant, the rate of hydroperoxides formation is related to the ratio of the lipid [LH] concentration to that of the antioxidant [M_iOH] concentration.

The classical rate laws for both uninhibited (R_c) and inhibited (R_A) lipid autoxidation, assuming the long chain approximation, are given by Eqs 1 and 2:

Rate of non-inhibited oxidation (R_c) $R_c=k_p [LH](R_{IN}/k_t)^{0.5}$ (1)

Rate of inhibited oxidation (R_{Am}) $R_{Am}=k_p[LH]R_{IN}/nk_{Am}[M_iOH]_0$ (2)

Inhibiting reactions, responsible to the antioxidant activity of the dimer $D_i(OH)_2$

Chain termination with H-atom transfer reaction:

$D_i(OH)_2+LO_2^\bullet \rightarrow D_i(OH)O^\bullet+LOOH$ (k_{Ad}) – the key reaction of inhibited lipid autoxidation

$D_i(OH)O^\bullet+LO_2^\bullet \rightarrow D_i(O^\bullet)_2+LOOH$ (k'_{Ad}) [$D_i(O^\bullet)_2$ –biradical of $D_i(OH)_2$; active intermediate]

Chain termination with cross recombination reaction:

$D_i(OH)O^\bullet + LO_2^\bullet \rightarrow D_i(OH)OL+O_2$ (active intermediate with one free OH group)

$D_i(O^\bullet)_2 + 2LO_2^\bullet \rightarrow$ inactive products (k''_{Ad})

Chain termination with homo recombination reaction:

2 $D_i(OH)O^\bullet \rightarrow [D_i(OH)O]_2$ - active intermediate with 2 free OH groups

Additional chain termination with H atom transfer reaction:

$D_i(OH)OL+LO_2^\bullet \rightarrow LOOH + D_i(O^\bullet)OL$ - active intermediate

$[D_i(OH)O]_2 + 2LO_2^\bullet \rightarrow 2LOOH +$ inactive products

Additional cross-recombination reaction:

$D_i(O^\bullet)OL +LO_2^\bullet \rightarrow$ inactive product

Additional homo-recombination reaction:

2 $D_i(O^\bullet)OL \rightarrow$ inactive product

Inhibited autoxidation - in presence of a biphenolic antioxidant $Q(OH)_2$

Termination (HAT) key reaction (k_{Aq})	$LO_2^\bullet + Q(OH)_2 \rightarrow LOOH + Q(OH)O^\bullet$	
Termination (Cross-dissproportionation)	$LO_2^\bullet + Q(OH)O^\bullet \rightarrow LOOH + Q(O)_2$	(k'_{Aq})
Termination (Homo-recombination)	$2Q(OH)O^\bullet \rightarrow$ Dimers	(k_{Rq})
Termination (Homo-dissproportionation) (k_{Dq})	$Q(OH)O^\bullet \rightarrow Q(OH)_2 + Q(O)_2$	

Scheme S2. Basic kinetic scheme of uninhibited and inhibited (in presence of monomers and dimers) lipid autoxidation.

Rate of inhibited oxidation (R_{Ad}): $R_{Ad}=k_p[LH]R_{IN}/nk_{Ad}[D_i(OH)_2]_0$ (3)

In Eqs 3-5: $k_p/(2k_t)^{0.5}$ is the oxidizability of the lipid substrate (linoleic acid glycerol ester); n- is the number of radicals trapped per inhibitor for molecule (the stoichiometric factor).

3.3.Determination of the main kinetic parameters of the studied compounds

Antioxidant efficiency means the potency of an antioxidant to increase the persistence towards oxidation of a lipid substrate by blocking the radical-chain

process. It was presented with a protection factor (PF) meaning how many times the antioxidant increases the persistence against oxidation of the lipid sample, determined as a ratio between the induction periods in presence (IP or IP_A) and in absence (IP_C) of an antioxidant, i.e. PF = IP_{Am}/IP_C for the monomers and PF = IP_{Ad}/IP_C for the dimers.

Inhibition degree (ID) is a measure of the antioxidant reactivity, which manifests how many times the antioxidant shortens the oxidation chain length, i.e. ID = R_C/R_{Am} in the case of monomers and ID = R_C/R_{Ad} in the case of dimers. For that reason, it is one of the most important kinetic parameters. Initial rates of lipid autoxidation in the absence (R_C) and in the presence of the antioxidant (R_{Am} or R_{Ad}) were found from the tangent at the initial phase of the kinetic curves of hydroperoxides accumulation.

Chain length of non-inhibited oxidation (ν_0) $\nu_c = R_c/R_{IN}$
Chain length of inhibited oxidation (ν_{Am} or ν_{Ad}) $\nu_{Am} = R_{Am}/R_{IN}$ and $\nu_{Ad} = R_{Ad}/R_{IN}$
Inhibition degree (ID)
In case of monomers ID = $R_c/R_{Am} = \nu_c/\nu_{Am}$
In case of dimers ID = $R_c/R_{Ad} = \nu_c/\nu_{Ad}$

4. Estimation of rate constant of antioxidant reaction with peroxyl radicals (k_A) using kinetic chemiluminescence (CL) method

$Y \rightarrow 2Y^\bullet$ (R_{IN})
$Y^\bullet + O_2 \rightarrow YO_2^\bullet$
$YO_2^\bullet + RH \rightarrow YOOH + R^\bullet$
$R^\bullet + O_2 \rightarrow RO_2^\bullet$
$RO_2^\bullet + RH \rightarrow ROOH + R^\bullet$ (k_p)
$2RO_2^\bullet \rightarrow R{=}O^* + ROH + O_2$ ($2k_t$)
$RO_2^\bullet + M_iOH \rightarrow ROOH + M_iO^\bullet$ (k_{Am}) in case of monomers
$RO_2^\bullet + M_iO^\bullet \rightarrow$ *inactive products* (k'_{Am})
$RO_2^\bullet + D_i(OH)_2 \rightarrow ROOH + D_i(O^\bullet)OH$ (k_{Ad}) in case of dimers
$RO_2^\bullet + D_i(O^\bullet)OH \rightarrow ROOH + D_i(O^\bullet)_2$ (k'_{Ad})
$2RO_2^\bullet + D_i(O^\bullet)_2 \rightarrow$ *inactive products* (k''_{Ad})

Scheme S3. Basic kinetic scheme of initiated oxidation of ethylbenzene (RH) in CL, in absence and in presence of studied compounds.

The CL assay for antioxidant activity is based on the competition between the self reaction of peroxy radicals (reaction step "k_t" in Scheme S2), giving rise to light emission, and scavenging the peroxy radicals by antioxidants (MOH), thus inhibiting the oxidation and thereby quenching the chemiluminescence [7]. The kinetic analysis of the reaction sequence (Scheme S3) affords convenient expressions relating the time profile of the chemiluminescence signal with the pertinent characteristics of an antioxidant being studied. The *strength* of an antioxidant is quantified by the rate constant k_A, whose value may be acquired from the slope of the chemiluminescence

time profile at the inflection point according to equation 4 [7] in which i_{rel} is a dimensionless light intensity given by the

$$(\mathrm{d}i_{\mathrm{rel}}/\mathrm{d}t)_{\max} = 0.237(k_A/(2k_t)^{0.5})R_{IN} \quad (4)$$

ratio of the intensities in the presence (I) and in the absence (I_0) of an antioxidant, $i_{\mathrm{rel}} = I/I_0$. It should be stressed that the consumption of an antioxidant in the chemiluminescence sample solution after the induction period results in recovery of the light-emission intensity, I_∞, i.e., $I_\infty = I_0$ and $i_{\mathrm{rel}} = I/I_0 = I/I_\infty$, and R_{IN} stays for the reaction initiation rate defined by equation 5. In the latter expression, k_{dec} is the rate constant of the initiator (Y) decomposition

$$R_{IN} = 2\gamma_c k_{\mathrm{dec}}[\mathrm{Y}] \quad (5)$$

to generate initiating radicals ($\mathrm{Y}^\bullet$ in Scheme S3) and γ_c is their probability to escape the solvent cage. The $2\gamma_c k_{\mathrm{dec}}$ data are available for most of "standard" initiators (such as AIBN, for instance) in a number of organic solvents and in a temperature range of 20 to 80 °C. The $2\gamma_c k_{\mathrm{dec}}$ data are available for most of "standard" initiators (such as AIBN, for instance) in a number of organic solvents and in a temperature range of 20 to 80 °C. Calculation of k_A from Eq. 4. was made taking into account that the $2k_t$ value obtained experimentally at the same conditions (chemiluminescence of ethylenbenzene) and temperature of 50 °C is equal to $2k_t = (1.90 \pm 0.05)\ 10^7\ \mathrm{M^{-1}s^{-1}}$ [7].

Table S2. Effect of the 10-fold higher concentration of the studied antioxidants (0.1 mM and 1.0 mM). For monomers (M) $PF^M_{10}=PF_1/PF_{0.1}$ and $ID^M_{10}=PF_1/PF_{0.1}$, for dimers (D) $PF^D_{10} = PF_1/PF_{0.1}$ and $ID^D_{10} = ID_1/PF_{0.1}$

Monomer	PF^M_{10}	ID^M_{10}	PF^D_{10}	ID^D_{10}	Dimer
**Cr**	1.3	1.1	1.4	1.5	**DCr**
**Va**	1.2	1.0	n.s.	n.s.	**DVa**
**Apo**	1.2	1.3	n.s.	n.s.	**DApo**
**Eu**	1.3	1.2	n.s.	n.s.	**DEu**
**iso-Eu**	1.8	1.3	n.d.	n.d.	**DisoEu**
**FA**	1.6	2.8	1.8	2.3	**DFA**
**HFA**	1.6	2.8	1.7	2.9	**DHFA**
**M1**	3.5	6.3	5.2	13.9	**D1**
**M2**	3.2	5.5	3.6	5.5	**D2**
**M3**	3.0	3.2	4.4	3.5	**D3**
**M4**	2.0	4.0	3.0	5.0	**D4**
**M5**	3.0	2.2	3.0	3.7	**D5**
**M6**	n.d.	n.d.	n.d.	n.d.	**D6**
**M7**	n.d.	n.d.			
**M8**	2.9	4.7	4.1	3.0	**D8**
**M9**	2.2	3.4	4.1	5.4	**D9**

Curcumin	4.5	6.7	-	-	-

n.d. – not determined, n.s. not soluble in lipid substrate.

M6, M7, D6, D7, DisoEu were studied only by theoretical methods.

Table S3. Effect of biphenyl structure.

Dimer/Monomer		Conc. mM	$PF_{D/M}$	$ID_{D/M}$
**D1/M1**		0.1	2.6	2.9
		1.0	3.9	4.6
**D2/M2**		0.1	1.5	1.6
		1.0	1.7	1.6
**D3/M3**		0.1	1.5	1.0
		1.0	3.7	1.1
**D4/M4**		0.1	1.1	1.0
		1.0	1.7	1.2
**D5/M5**		0.1	1.0	2.4
		1.0	1.0	1.4
**D8/M8**		0.1	1.0	0.7
		1.0	1.3	0.7
**D9/M9**		0.1	1.0	0.7
		1.0	1.6	1.1
**DFA/FA**		0.1	1.0	1.0
		1.0	1.0	1.0
**DCr/Cr**		0.1	0.9	0.6
		1.0	2.0	1.0
**Curc/M1**		0.1	1.6	4.4
		1.0	3.9	4.7
**Curc/M3**		0.1	1.6	1.4
		1.0	2.4	3.0

5. DFT computational studies

Table S4. B3LYP/6-31+G(d,p) gas phase calculated enthalpies H_{298} (a.u.) and dihedral angles (°) for AO structures shown in Figure 1A-C and respective radicals (AO•). BDEs (kcal mol^{-1}) are derived from H_{298} values calculated for neutral (AO) and radical (AO•, H•) species.

**Compound**	**H_{298} (AO)**	**H_{298} (AO•)**	**BDE**	**θ ° (AO)**	**θ ° (AO•)**	Also mentioned in:
**Curcumin**	-1263.272328	-1262.646509	78.8			[8]

**Cr**	-461.169138	-460.541707	79.8			[9]
**Va**	-535.200734	-534.567085	83.7			[9]
**Apo**	-574.496592	-573.863718	83.2			[9]
**Eu**	-538.531135	-537.903146	80.1			[9]
**isoEu**	-538.541134	-537.917493	77.4			[9]
**FA**	-687.821912	-687.193184	80.6			[10]
**M1**	-1302.554493	-1301.928076	79.2			[10]
**M3**	-1032.317391	-1031.689946	79.8			
**M6**	-804.486573	-803.859376	79.6			
**M7**	-843.773289	-843.146109	79.9			
**M2**	-653.066142	-652.438022	80.2			[10]
**HFA**	-689.020063	-688.391341	80.6			
**M4**	-805.682054	-805.054012	80.2			
**M5**	-997.374041	-996.745838	80.3			
**M8**	-1036.666595	-1036.038505	80.2			[11]
**M9**	-1154.525497	-1153.897424	80.2			[11]
**HCh**	-499.257728	-498.641117	73.0			
**HPh**	-612.594197	-611.975469	74.3			
**CA**	-648.548130	-647.928078	75.2			[12]
**HCA**	-649.745959	-649.129776	72.7			
**DCr**	-921.154915	-920.528642	79.1	62.9	61.1	[9]
**DVa**	-1069.218727	-1068.586389	82.9	62.7	59.5	[9]
**DApo**	-1147.810582	-1147.178797	82.5	63.0	60.4	[9]
**DEu**	-1075.878884	-1075.252057	79.4	63.0	61.1	[9]
**DisoEu**	-1075.899195	-1075.276325	76.9	63.5	62.9	[9]
**DFa**	-1374.461072	-1373.833542	79.9	63.1	60.3	[10],[12]
**D1**	-1302.554462	-1301.928383	78.9	63.6	60.5	[10],[12]
**D3**	-2063.452014	-2062.825554	79.2	63.0	61.7	
**D6**	-1607.790268	-1607.164203	78.9	63.0	60.6	
**D7**	-1686.363704	-1685.737657	78.9	62.8	60.6	
**D2**	-1304.948860	-1304.321729	79.6	62.2	59.7	[10]
**DHFA**	-1376.853281	-1376.225057	80.3	63.6	60.6	
**D4**	-1610.180433	-1609.553451	79.5	63.9	60.5	
**D5**	-1993.565020	-1992.938034	79.5	63.1	61.9	
**D8**	-2072.150177	-2071.523306	79.4	62.3	60.9	[11]
**D9**	-2307.867988	-2307.867988	79.6	63.2	61.5	[11]
**DHCh**	-997.331996	-996.716674	72.2	62.8	61.3	
**DHPh**	-1224.005687	-1223.387443	74.0	63.2	62.0	
**DCA**	-1295.913311	-1295.293777	74.8	62.5	60.8	
**DHCA**	-1298.307686	-1297.691985	72.4	62.6	61.1	

References

[1] Feng J-Y, Liu Z-Q. Feruloylacetone as the model compound of half-curcumin: synthesis and antioxidant properties. Eur J Med Chem 2011;46:1198–206. https://doi.org/10.1016/j.ejmech.2011.01.039.

[2] Typek R, Dawidowicz AL, Bernacik K, Stankevič M. Feruloyloacetone can be the main curcumin transformation product. Food Chem 2019;286:136–40. https://doi.org/10.1016/j.foodchem.2019.01.194.

[3] Popov, A.; Yanishlieva N. S., J Compt Rend Acad Bulg Sci 1968;21:443–6.

[4] Christie WW, Han X. Lipid Analysis. 4th ed. Woodhead Publishing; 2012.

[5] Yanishlieva, N. V.; Popov, A. D.; Marinova E. M., Compt Rend Acad Bulg Sci 1978;31:869–71.

[6] Kancheva VD. Phenolic antioxidants – radical-scavenging and chain-breaking activity: A comparative study. Eur J Lipid Sci Technol 2009;111:1072–89. https://doi.org/10.1002/ejlt.200900005.

[7] Fedorova GF, Menshov VA, Trofimov A V, Vasil'ev RF. Facile chemiluminescence assay for antioxidative properties of vegetable lipids: fundamentals and illustrative examples. Analyst 2009;134:2128–34. https://doi.org/10.1039/B905059K.

[8] Slavova-Kazakova AK, Angelova SE, Veprintsev TL, Denev P, Fabbri D, Dettori MA, et al. Antioxidant potential of curcumin-related compounds studied by chemiluminescence kinetics, chain-breaking efficiencies, scavenging activity (ORAC) and DFT calculations. Beilstein J Org Chem 2015;11. https://doi.org/10.3762/bjoc.11.151.

[9] Koleva, L.; Angelova, S.; Dettori, M. A.; Fabbri, D.; Delogu, G.; Kancheva VD. Antioxidant activity of selected o-Antioxidant activity of selected o-methoxyphenols and biphenols: theoretical and experimental studies. Bulg Chem Comm 2018;50:238–46.

[10] Kancheva, V. D.; Slavova-Kazakova, A.; Fabbri, F.; Angelova, S.; Dettori, M. A.; Jordan Nechev, Delogu, G., Antiradical and Antioxidant Activities of New Natural-like Hydroxylated Biphenyls of Dehydrozingerone, Zingerone and Ferulic Acid. Comptes Rendus l'Académie Bulg Des Sci 2013;66:361–8.

[11] Slavova-Kazakova A, Angelova S, Fabbri D, Antonietta Dettori M, Kancheva VD, Delogu G. Antioxidant properties of novel curcumin analogues: A combined experimental and computational study. J Food Biochem 2021;45:e13584. https://doi.org/10.1111/jfbc.13584.

[12] Kancheva VD., Angelova SE. Synergistic Effects of Antioxidant Compositions during Inhibited Lipid Autoxidation. LIPID PEROXIDATION INHIBITION , EFFECTS AND MECHANISMS (ed.: Catala, A.), Nova Science Publishers, Inc., New York, 2016.
